# A Synergistic Approach with Doxycycline and Spirulina Extracts in DNBS-Induced Colitis: Enhancing Remission and Controlling Relapse

**DOI:** 10.3390/jox15050160

**Published:** 2025-10-03

**Authors:** Meriem Aziez, Mohamed Malik Mahdjoub, Tahar Benayad, Ferroudja Abbas, Sarah Hamid, Hamza Moussa, Ibrahima Mamadou Sall, Hichem Tahraoui, Abdeltif Amrane, Noureddine Bribi

**Affiliations:** 1 Laboratoire de Biotechnologie Végétale et Ethnobotanique, Faculté des Sciences de la Nature et de la Vie, Université de Bejaia, Bejaia 06000, Algeria; meriem.aziez@univ-bejaia.dz (M.A.); sarah.hamid@univ-bejaia.dz (S.H.); noureddine.bribi@univ-bejaia.dz (N.B.); 2 Département des Sciences de la Nature et de la Vie, Faculté des Sciences, Université d’Alger 1, Didouche Mourad, Alger 16000, Algeria; m.mahdjoub@univ-bouira.dz; 3 Département des Sciences Biologiques, Faculté des Sciences de la Nature et de la Vie et des Sciences de la Terre, Université de Bouira, Bouira 10000, Algeria; h.moussa@univ-bouira.dz; 4 Laboratoire Central de la Police Scientifique, Alger 16000, Algeria; taharbenayad1@gmail.com (T.B.); ferouab@yahoo.fr (F.A.); 5 Department of Anatomic Pathology, Faculty of Veterinary Medicine, University of Agricultural Sciences and Veterinary Medicine of Cluj-Napoca, 400372 Cluj-Napoca, Romania; ibrahima.sall@student.usamvcluj.ro; 6 Laboratory of Biomaterials and Transport Phenomena, University of Medea, Medea 26000, Algeria; hichemm.tahraouii@gmail.com; 7 Ecole Nationale Supérieure de Chimie de Rennes, Université de Rennes, CNRS, ISCR—UMR6226, F-35000 Rennes, France

**Keywords:** doxycycline, *Arthrospira platensis*, experimental colitis, synergistic therapy

## Abstract

**Background:** Chronic relapsing colitis involves immune dysregulation and oxidative stress, making monotherapies often insufficient. This study investigates a therapeutic strategy combining doxycycline (Dox), an immunomodulatory antibiotic, with *Arthrospira platensis* extracts to enhance anti-inflammatory and antioxidant effects, improving remission and controlling relapse. **Methods:** Ethanolic (ES) and aqueous (AS) extracts of *A. platensis* were chemically characterized by GC-MS after derivatization. Colitis was induced in mice using two intrarectal DNBS administrations spaced 7 days apart, with oral treatments (Dox, ES, AS, or combinations) given daily between doses. Disease progression was evaluated through clinical monitoring, histological scoring, and biochemical analysis, including MPO and CAT activities, as well as NO, MDA, and GSH levels. **Results:** GC-MS identified 16 bioactive compounds in each extract. ES contained mainly fatty acids and amino acids, whereas AS was rich in polysaccharides and phytol. Combined doxycycline and *A. platensis* extracts significantly enhanced recovery in reactivated DNBS colitis compared to monotherapies. Each treatment alone reduced disease severity, but their combination showed synergistic effects, significantly reducing disease activity index (*p* < 0.001), restoring mucosal integrity, and modulating inflammatory and oxidative markers (*p* < 0.001). **Conclusion:** Doxycycline potentiates the anti-colitic effects of *A. platensis* extracts via complementary mechanisms, offering a promising combination for managing relapsing colitis.

## 1. Introduction

Inflammatory bowel diseases (IBDs), including Crohn’s disease and ulcerative colitis, are chronic and recurrent inflammatory disorders of the gastrointestinal tract [1,2]. Their pathogenesis is multifactorial and involves dysregulated immune responses, excessive oxidative and nitrosative stress, epithelial barrier dysfunction, and gut microbiota imbalance [3,4]. These interconnected mechanisms contribute to the onset and persistence of intestinal inflammation and tissue injury [5]. Current therapeutic strategies, such as aminosalicylates, corticosteroids, immunosuppressants, and biologics targeting specific cytokines, focus mainly on immune modulation [6,7]. Although these treatments can induce remission in some patients, they often show limited long-term efficacy, risk of relapse, and potential adverse effects [8,9]. Moreover, they do not directly address oxidative stress or microbiota dysbiosis, which are central contributors to disease progression [10,11]. This highlights the need for complementary or combination therapies capable of targeting multiple pathological pathways to achieve more effective and durable disease control [12,13].

In this context, doxycycline, an established member of the tetracycline class, has garnered attention beyond its conventional antimicrobial role [14,15,16]. Recognized as a prototypical xenobiotic, doxycycline exhibits a wide spectrum of biological activities that include anti-inflammatory, antioxidant, and matrix metalloproteinase (MMP) inhibitory effects [16,17,18]. These pleiotropic properties have led to its repurposing in various inflammatory and degenerative diseases, including models of acute and chronic colitis [16,19,20]. Doxycycline has been shown to attenuate intestinal inflammation by modulating the nuclear factor kappa B (NF-κB) signaling, reducing oxidative stress, and preserving mucosal integrity [21,22]. However, prolonged use is often limited by concerns over microbiota disruption, antibiotic resistance, and systemic toxicity [23,24]. These limitations underline the need for safer and more sustainable approaches, such as combination therapies that optimize the therapeutic benefit while minimizing adverse effects.

To address these limitations, growing interest has been directed toward natural bioactive compounds with anti-inflammatory and antioxidant potential, such as those derived from *Arthrospira platensis* (commonly known as spirulina) [25]. This cyanobacterium is rich in functional molecules, including phycocyanin, polysaccharides, polyunsaturated fatty acids, phenolic acids, and essential amino acids [26,27], that have demonstrated anti-inflammatory, antioxidant, antimicrobial, and tissue-protective activities in various preclinical models [28,29,30]. Several studies have reported that both aqueous and ethanolic extracts of spirulina can alleviate symptoms of chemically induced colitis by reducing oxidative damage, preserving intestinal barrier function, and modulating cytokine expression [28,31,32]. Importantly, spirulina is considered a safe, food-grade xenobiotic with high biocompatibility, making it a promising candidate for integrative strategies aimed at reducing drug dependency and enhancing therapeutic outcomes in chronic inflammatory diseases [33,34].

To the best of our knowledge, tetracycline antibiotics, particularly minocycline and doxycycline, have been independently evaluated in various models of acute and chronic colitis, primarily due to their dual anti-inflammatory and antimicrobial properties [21,35,36]. Similarly, several studies have explored the protective effects of *A. platensis* and its bioactive extracts in experimental colitis, highlighting their ability to mitigate oxidative stress and mucosal damage [28,31,32,37]. However, the potential therapeutic benefit of combining doxycycline with spirulina extracts has not yet been investigated in a model that mimics the recurrent and relapsing nature of human IBD. This gap is particularly relevant given the complementary mechanisms of action of both agents, which may enhance overall efficacy when used in combination.

To address the complexity of IBD pathogenesis, this study investigated a dual therapeutic strategy combining *A. platensis* extracts with the synthetic agent doxycycline. Specifically, we evaluated the effects of ethanolic (ES) and aqueous (AS) extracts, alone or in combination with doxycycline, in a relapsing colitis model induced by 2,4-dinitrobenzenesulfonic acid (DNBS) in mice. To support the interpretation of the biological responses, the extracts were chemically characterized using Gas Chromatography–Mass Spectrometry (GC–MS) after derivatization, enabling a comprehensive profiling of their bioactive constituents. This dual approach aimed to determine whether the combination therapy could provide superior protection and promote sustained remission compared to monotherapies by targeting multiple pathological pathways involved in IBD relapse.

## 2. Materials and Methods

### 2.1. Biological Material and Reagents

Dry *A. platensis* biomass was obtained from a controlled algal culture at Kasdi Merbah University, Ouargla, Algeria. All chemicals and reagents were sourced from Sigma-Aldrich (Madrid, Spain), unless otherwise specified.

### 2.2. Preparation of *A. platensis* Extracts

*A. platensis* powder was extracted using either ethanol (80%) or distilled water at a 1:10 (*w*/*v*) ratio, under continuous stirring for 24 h at 25 °C in the dark. The mixtures were filtered through Whatman No. 1 filter paper, and the resulting ES and AS extracts were dried at 40 °C and stored at 4 °C for further analysis.

### 2.3. Derivatized GC-MS Profiling

For the GC-MS analysis, the ES and AS extracts were derivatized using N-methyl-N-(trimethylsilyl)-trifluoroacetamide (MSTFA) according to the method of Morsy et al. [28]. Analyses were performed on a PerkinElmer Clarus 500 gas chromatograph (PerkinElmer, Waltham, MA, USA) coupled to a quadrupole mass spectrometer (mass range: *m*/*z* 20–550; source temperature: 250 °C). Separation was achieved on an Elite 5 MS capillary column (30 m × 0.25 mm internal diameter × 0.25 µm film thickness) with helium as the carrier gas at a flow rate of 1 mL/min. The oven temperature was initially set at 70 °C for 4 min, increased at a rate of 4 °C/min to reach 220 °C, and held for 15 min. The total run time was 56.5 min. A 1 µL aliquot of each derivatized extract was injected, with an ionization energy of 70 eV. Compound identification was carried out by matching the mass spectra and retention indices of the detected peaks with reference standards from the NIST and WILEY mass spectral libraries.

### 2.4. Animals and Experimental Design

A total of forty-nine female BALB/c mice (20–25 g), obtained from the Pasteur Institute (Algiers, Algeria), were housed under standard conditions with a 12 h light/dark cycle and free access to food and water. All procedures were approved by the Ethics Committee of the LBVE Laboratory (Ref. CE-LBVE-2024-117) and conducted in accordance with EU Directive 2010/63/EU.

**Figure 1:**
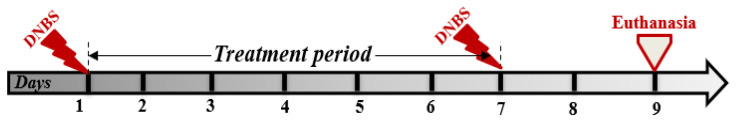
Experimental design of the DNBS-induced relapsing colitis model in BALB/c mice, treated with multiple oral administrations of ethanolic (ES) and aqueous (AS) extracts of *Arthrospira platensis*, alone or in combination with doxycycline.

Mice were randomly assigned to seven groups (*n* = 7 per group): one non-colitic control group and six colitic groups. Among the colitic groups, one remained untreated, while the other five received oral treatments as follows: doxycycline (25 mg/kg), ES extract (100 mg/kg), AS extract (100 mg/kg), and combinations of doxycycline with either ES or AS. Relapsing colitis was induced by two intrarectal administrations of DNBS (250 mg/kg in 0.1 mL of 50% ethanol) under light anesthesia as previously described by T. T. Hove et al. [38]. The first instillation was performed on day 1, immediately followed by the initiation of daily oral treatments (doxycycline, ES extract, AS extract, or their combinations), which continued once daily through day 7. On day 7, a second DNBS instillation was administered to trigger relapse, and animals were euthanized on day 9 for endpoint analyses (Figure 1).

### 2.5. Clinical and Macroscopic Evaluation of Colitis

During the experiment, each clinical symptom observed in the animals (wet anus, bleeding, altered stool, piloerection, hypoactivity) was scored as 1 point. Weight loss was scored separately, with 1 point assigned for a 5–10% loss and 2 points for a loss greater than 10% in 24 h. The total clinical score reflected disease severity. At the end of the experimental period, animals were euthanized and dissected. The entire colon was removed, weighed, and its length was measured to calculate the weight-to-length ratio.

### 2.6. Histopathological Assessment

A small portion of colon specimens was fixed in 4% buffered formaldehyde for the histological study. Cross-sections were selected and embedded in paraffin, and subsequently, 5 µm full-thickness sections were taken at different levels, mounted on silane-coated glass slides, and stained with hematoxylin and eosin (H&E) for histological scoring and observed with a Leica microscope. Histological scoring of colonic tissues was performed based on inflammation, mucosal ulceration, and necrosis. Ulceration was graded on a 0–4 scale: 0 = absent; 1 = mild (0–25% mucosal surface affected); 2 = moderate (25–50%); 3 = severe (50–75%); 4 = extensive/full thickness (>75%). Microscopic changes were similarly scored for severity (0 = none, 1 = minimal, 2 = mild, 3 = moderate, 4 = severe).

### 2.7. Assessment of Inflammatory and Redox Markers

To assess the inflammatory and redox alterations induced by DNBS and the potential protective effects of *A. platensis* extracts, several biochemical markers were quantified in the colonic tissues. Colonic tissue samples were homogenized in phosphate buffer (50 mM, pH 7.4) and centrifuged to remove debris and obtain the post-mitochondrial supernatant (PMS), which was used for biochemical assays.

#### 2.7.1. Myeloperoxidase (MPO) Activity

MPO activity was quantified according to the method described by Merakeb et al. [39]. The assay mixture consisted of 50 mM phosphate buffer (pH 6.0), 0.167 mg/mL o-dianisidine hydrochloride, 0.0005% hydrogen peroxide, and PMS. The change in absorbance was monitored at 460 nm over 3 min using a microplate reader. MPO activity was calculated using the molar extinction coefficient of 11.3 mM^−1^·cm^−1^ to convert absorbance changes into the rate of H_2_O_2_ decomposition. The results are expressed as mM of H_2_O_2_ decomposed per minute per gram of colon (wet weight), allowing a quantitative and physiologically relevant measure of neutrophil infiltration and inflammation.

#### 2.7.2. Nitrite Quantification

Nitrite concentrations, reflecting NO production, were determined by the Griess reaction [40]. Briefly, PMS samples were deproteinized by adding an equal volume of 10% trichloroacetic acid (TCA), followed by centrifugation at 1500× *g* for 10 min at 4 °C. The supernatant was mixed with an equal volume of Griess reagent and incubated in the dark at room temperature for 20 min. Absorbance was measured at 543 nm, and nitrite concentrations were determined by reference to a sodium nitrite standard curve (ranging from 1 to 128 μM). Results were expressed as µM of nitrite per 100 mg of tissue.

#### 2.7.3. Measurement of Lipid Peroxidation

Lipid peroxidation was assessed by quantifying malondialdehyde (MDA) levels using the thiobarbituric acid reactive substances (TBARS) assay [31]. PMS samples were mixed with 35% TCA (1:1, *v*/*v*), incubated on ice for 1 h, and centrifuged at 1500× *g* for 10 min at 4 °C. The supernatant was reacted with 0.8% thiobarbituric acid in acetic acid, heated at 95 °C for 60 min, then cooled on ice. The pink chromogen formed was measured at 532 nm. MDA concentrations were calculated using the extinction coefficient of 1.56 × 10^5^ M^−1^.cm^−1^ and expressed as µM per gram of wet tissue.

#### 2.7.4. Catalase (CAT) Activity

Catalase activity was determined by measuring the rate of decomposition of hydrogen peroxide at 240 nm, as described by Avula et al. [41]. The assay mixture contained 1.95 mL of 0.019 M hydrogen peroxide in phosphate buffer (50 mM, pH 7.0) and 50 µL of PMS. The decrease in absorbance at 240 nm was recorded every 30 s for 3 min at 25 °C. Catalase activity was calculated using the molar extinction coefficient of 43.6 M^−1^·cm^−1^ for H_2_O_2_ and expressed as mM H_2_O_2_ degraded per minute per gram of tissue.

#### 2.7.5. Reduced Glutathione (GSH) Content

GSH content was measured by the described method of Rathore et al. [42]. Briefly, PMS samples were deproteinized by mixing with an equal volume of 4% sulfosalicylic acid, followed by centrifugation at 1500× *g* for 10 min at 4 °C. The supernatant was added to a reaction mixture containing 0.1 M phosphate buffer (pH 7.4) and 0.4% of 5,5′-dithiobis-(2-nitrobenzoic acid) (DTNB). The absorbance of the yellow-colored 5-thio-2-nitrobenzoic acid (TNB) product was measured at 412 nm within 5 min. GSH concentrations were quantified by reference to a standard curve constructed with known concentrations of reduced glutathione and expressed as µM per 100 mg of protein. Total protein content in PMS was determined using the Bradford method, with bovine serum albumin as the standard (ranging from 0 to 100 µg/mL).

### 2.8. Statistical Analysis

Data are presented as mean ± standard deviation (SD). Statistical analyses were performed using GraphPad Prism 8.0.2.263 (GraphPad Software, San Diego, CA, USA). Group comparisons were conducted using one-way ANOVA, followed by Dunnett’s test; A *p*-value < 0.05 was considered statistically significant. In addition, Two-way ANOVA was used to evaluate potential synergistic interactions between doxycycline and *A. platensis* extracts, with effect sizes and 95% CIs reported alongside *p*-values.

## 3. Results

### 3.1. GC-MS Analysis of Derivatized *A. platensis* Extracts

Following the derivatization of ES and AS extracts, the chemical composition of the samples was analyzed using GC-MS. The retention times of the major compounds identified in the ES and AS extracts are presented in Supplementary Figures S1 and S2, respectively. At the same time, their relative abundances (expressed as area percentages) and molecular formulas, and additional identification criteria are detailed in Supplementary Tables S1 and S2.

The GC-MS analysis of the ES extract revealed a complex mixture primarily composed of fatty acids and amino acids. A total of sixteen distinct compounds were identified, with Palmitic acid (18.07%), 2-O-Glycerol-α-D-galactopyranoside (16.32%), L-Methionine (13.62%), and D-phenylalanine (11.79%) as the major constituents. In contrast, the AS extract exhibited a distinct phytochemical profile, dominated by polysaccharides and terpenoid compounds. Sixteen compounds were detected, including D-Xylopyranose (14.67%), Phytol (11.23%), α,α’-Trehalose (8.08%), and Palmitic acid (7.93%) as the most abundant.

### 3.2. Clinical Assessment of Colitis Severity

**Figure 2:**
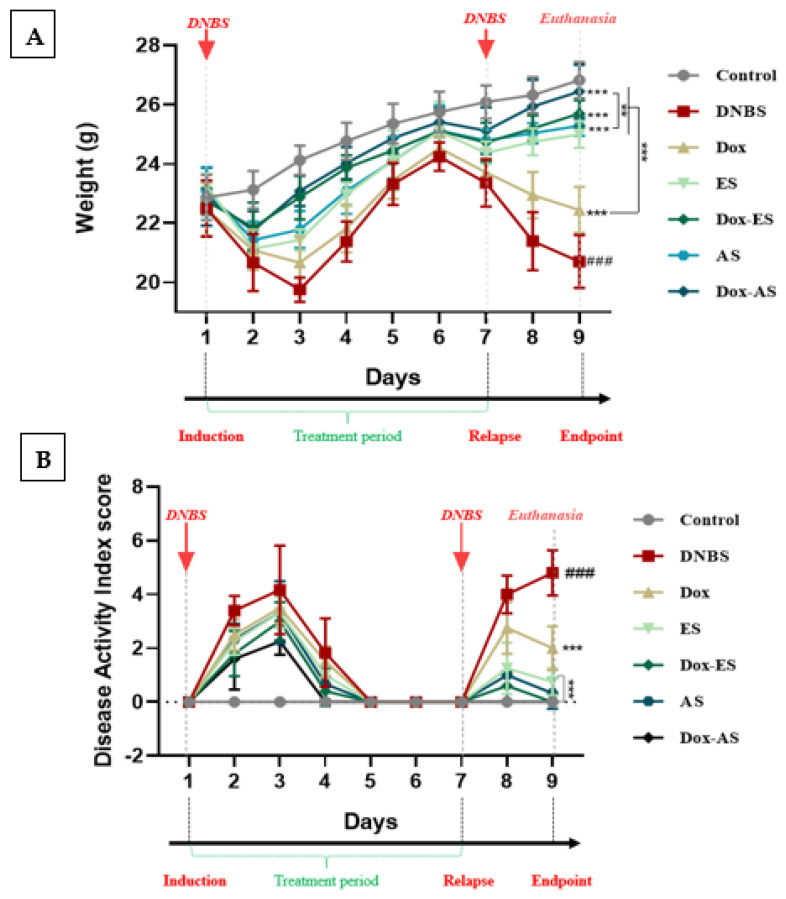
Clinical evaluation of *A. platensis* extracts (ES and AS), alone or combined with doxycycline (Dox), on DNBS-induced colitis in BALB/c mice. (**A**) Body weight evolution; (**B**) Disease Activity Index (DAI) during acute and relapse phases. The experimental timeline is indicated above the graphs, showing DNBS administrations (days 1 and 7), the treatment period (days 1–7), and euthanasia (day 9). Data are presented as mean ± standard deviation (SD) (*n* = 7 per group). Statistical analysis was performed using one-way ANOVA followed by Dunnett’s multiple comparisons test. ** *p* < 0.01 and *** *p* < 0.001 vs. the DNBS group; ### *p* < 0.001 vs. the control group.

To investigate the clinical efficacy of *A. platensis* extracts (ES and AS), alone or in combination with doxycycline, both body weight evolution and the DAI were monitored as sensitive and early indicators of colitis severity and overall health deterioration in murine models, providing a comprehensive evaluation of inflammation-induced damage and therapeutic response (Figure 2A,B). The DAI incorporated multiple clinical parameters, including body weight variation, stool consistency, rectal bleeding, piloerection, and reduced activity, offering a composite and objective measure of disease progression during both acute and relapse phases.

In this study, weight changes were carefully monitored across the experimental timeline, with comparative analyses explicitly performed on day 3 and day 9, corresponding to 48 h after each DNBS induction. A significant decrease in body weight was observed in the untreated colitic group compared to the healthy control group at both time points (*** *p* < 0.001), reflecting severe physical deterioration likely associated with intestinal dysfunction and mucosal inflammation. At day 3, mice treated with *A. platensis* extracts (ES or AS), alone or in combination with doxycycline (Dox-ES or Dox-AS), exhibited significant protection against weight loss (*** *p* < 0.001), while the doxycycline monotherapy group showed no statistically significant improvement (*p* > 0.05). At day 9, all treatment groups, including doxycycline alone, showed a significant improvement compared to the untreated colitic group (*** *p* < 0.001). These data reinforce the relevance of body weight as a clinical marker and underscore the beneficial impact of *A. platensis* extracts, particularly in combination therapy.

Body weight evolution was integrated as a component in the composite DAI, together with stool consistency, rectal bleeding, and physical activity. A significant and progressive increase in DAI was observed following the first DNBS administration in the untreated colitic group compared with the control group (*** *p* < 0.001), indicating the successful onset of acute intestinal inflammation. In contrast, treatment with *A. platensis* extracts, especially when combined with Doxycycline, resulted in a gradual and notable reduction in DAI values, suggesting a favorable impact on the attenuation of acute inflammation. After the second DNBS administration, intended to mimic colitis relapse, untreated colitic mice exhibited a significant and exacerbated increase in DAI scores compared to the non-colitic group, highlighting a failure in maintaining remission. In contrast, mice receiving either doxycycline or *A. platensis* extracts alone showed moderate improvement. At the same time, those treated with combinations displayed a significantly attenuated DAI increase during the relapse phase compared with the untreated colitis group (*p* < 0.001), with final DAI scores nearing those of the non-colitic control (*p* > 0.05). These results suggest a cumulative and potentially synergistic effect of the combined treatments in promoting remission and preventing colitis reactivation.

### 3.3. Macroscopic Assessment of Colonic Damage

**Figure 3:**
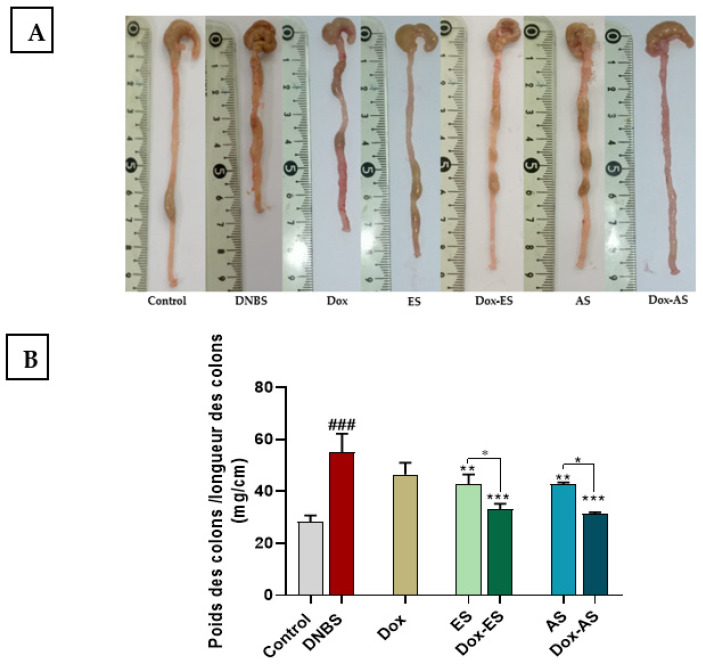
Effect of *A. platensis* extracts (ES and AS), alone or in combination with doxycycline (Dox), on macroscopic colonic damage in DNBS-induced acute and relapsing colitis in BALB/c mice. (**A**) Representative macroscopic appearance of colons; (**B**) Colon weight-to-length ratio. Data are expressed as mean ± standard deviation (SD) (*n* = 7 per group). Statistical analysis was performed using one-way ANOVA followed by Dunnett’s multiple comparisons test. * *p* < 0.05, ** *p* < 0.01, *** *p* < 0.001 vs. the DNBS group; ### *p* < 0.001 vs. the healthy control group.

The colon weight-to-length ratio, a robust indicator of inflammation-associated edema and tissue remodeling, was significantly elevated in DNBS-treated mice (55.02 ± 7.21) compared with the healthy control group (28.33 ± 2.40 mg/cm; *p* < 0.001), indicating pronounced structural alterations. Treatment with doxycycline alone yielded a moderate, non-significant reduction in this parameter (43.83 ± 4.63 mg/cm; *p* > 0.05), suggesting limited efficacy in mitigating structural colonic alterations. In contrast, administration of *A. platensis* extracts resulted in significant attenuation of colonic inflammation, as evidenced by reductions in the ratio to 42.75 ± 3.80 mg/cm for ES and 42.84 ± 0.68 mg/cm for AS, compared with the untreated colitic group (*p* < 0.01), indicating effective attenuation of colonic inflammation and edema. Importantly, combination therapies of doxycycline with either ES or AS produced a more pronounced decrease in the ratio (34.39 ± 4.33 and 33.92 ± 0.81 mg/cm, respectively) compared to the untreated colitic group (*p* < 0.001). Moreover, these decreases were significantly greater than those achieved by the corresponding extracts alone administered as monotherapy (*p* < 0.05). However, no significant interaction was observed between Dox and either extract for this parameter (Dox-ES *p* = 0.9474; Dox-AS *p* = 0.6701), indicating that the observed effects are primarily additive rather than truly synergistic (Figure 3A,B).

### 3.4. Histological Assessment of Colonic Tissue

**Figure 4:**
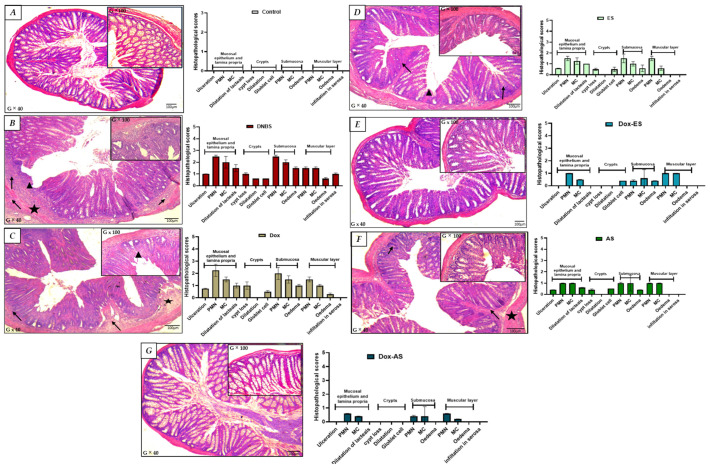
Histological evaluation of colonic tissues in DNBS-induced colitis and after treatment with doxycycline, ethanolic (ES) and aqueous (AS) extracts of *A. platensis*, alone or in combination. Representative hematoxylin–eosin-stained transverse sections of colonic tissues (G × 40 and G × 100) from: (**A**) control group showing preserved colonic architecture (no significant findings); (**B**) untreated colitic group, with multifocal infiltration of the mucosa and submucosa by polymorphonuclear (PMN) and mononuclear (MC) cells (➔), focal depletion of intestinal crypts, and focal mucosal ulceration (➤); (**C**) doxycycline-treated group exhibiting partial structural improvement; (**D**) group treated with ethanolic extract (ES) showing moderate tissue restoration; (**E**) group treated with aqueous extract (AS) showing similar improvements; (**F**) Dox + ES group demonstrating enhanced protection; and (**G**) Dox + AS group displaying nearly complete normalization of colonic histology. Key: (★) submucosal edema; (➔) infiltration of polymorphonuclear and mononuclear cells in the submucosa; (➤) crypt disruption and/or focal mucosal ulceration.

Histological analysis provided further confirmation of the protective effects of the treatments. As shown in Figure 4A, colon sections from healthy control mice exhibited a well-preserved mucosal architecture, with intact epithelial layers, clearly defined crypts, and no signs of edema or inflammatory infiltration. In contrast, microscopic examination of colonic samples from the untreated colitic group revealed significant histopathological alterations, characterized by multifocal infiltration of the mucosa and submucosa by polymorphonuclear and mononuclear cells, focal depletion of intestinal crypts, and localized ulceration of the mucosa (Figure 4B).

Treatment with doxycycline alone led to only modest improvements, with a slight reduction in edema and infiltration by polymorphonuclear and mononuclear cells; however, the overall architecture remained disrupted, with evident crypt distortion and partial epithelial damage. These findings suggest that doxycycline alone provided limited protection against DNBS-induced injury (Figure 4C). Conversely, treatment with ES (Figure 4D) or AS (Figure 4E) resulted in more pronounced improvements. Both treatments reduced edema and inflammatory infiltration by polymorphonuclear and mononuclear cells and partially restored mucosal architecture. Although the crypts remained slightly shortened or irregular in some areas, the epithelial continuity was largely recovered, supporting the anti-inflammatory properties of the extracts.

The combined treatments (Figure 4F,G) demonstrated superior protective effects. In particular, the Dox-AS group showed near-complete restoration of colonic architecture. The crypts were intact, epithelial integrity was restored, and no signs of inflammation or edema were detected. This structural normalization closely resembled that of the non-colitic control group, strongly supporting a synergistic interaction between doxycycline and *A. platensis* in alleviating DNBS-induced colonic damage.

### 3.5. MPO Activity as a Marker of Neutrophil Infiltration

**Figure 5:**
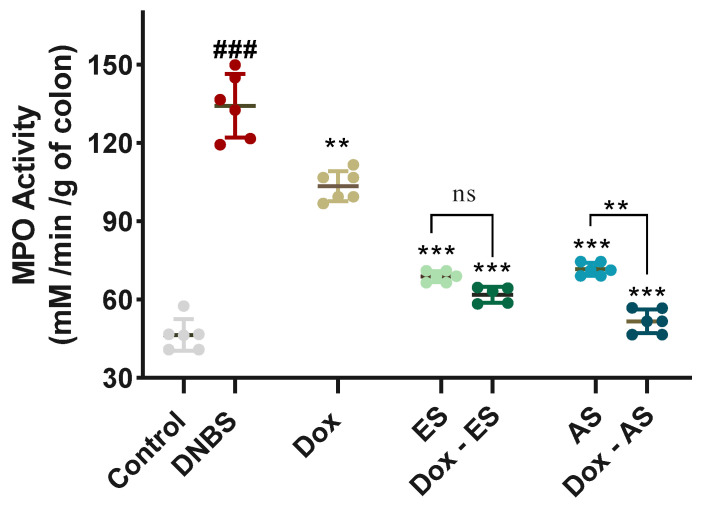
Effect of *A. platensis* extracts (ES and AS), alone or in combination with doxycycline (Dox), on colonic myeloperoxidase (MPO) activity (mM/min/g of colon) in DNBS-induced colitis in BALB/c mice. MPO activity was determined spectrophotometrically using o-dianisidine and H_2_O_2_ as substrates. Data are expressed as mean ± standard deviation (*n* = 7 per group). Statistical analysis was performed using one-way ANOVA followed by Dunnett’s multiple comparisons test. ns *p* > 0.05, ** *p* < 0.01, *** *p* < 0.001 vs. the DNBS group; ### *p* < 0.001 vs. the control group.

To further confirm the extent of neutrophilic infiltration observed histologically, colonic MPO activity was assessed as a reliable biochemical marker (Figure 5). The DNBS-induced colitic group exhibited a significant increase in MPO activity (134.18 ± 12.24 mM/min/g of colon) compared with the healthy control group (47.77 ± 7.00 mM/min/g of colon; *p* < 0.001), reflecting pronounced neutrophil infiltration and reactivated colonic inflammation. Treatment with doxycycline alone led to a significant decrease in MPO levels (103.60 ± 6.81 mM/min/g of colon) compared to the untreated colitic group (*p* < 0.01). However, the values remained elevated compared to the control group, indicating a partial anti-inflammatory effect. Both ES and AS extracts also produced significant reductions in MPO activity to 72.26 ± 14.14 and 65.90 ± 13.08 mM/min/g of colon, respectively, compared with the untreated colitic group (*p* < 0.001), supporting their anti-inflammatory potential. Notably, the combined treatments resulted in even greater reductions in MPO activity, with values of 61.82 ± 3.07 mM/min/g of colon for the Dox-ES group and 51.59 ± 5.09 mM/min/g of colon for the Dox-AS group. A significant interaction between Dox and ES (*p* = 0.0025), indicating a true synergistic effect for the Dox-ES combination, whereas no significant interaction was observed between Dox and AS (*p* = 0.0935), suggesting that the enhanced effect of Dox-AS is largely additive rather than synergistic.

### 3.6. Lipid Peroxidation and Nitrosative Stress Markers

**Figure 6:**
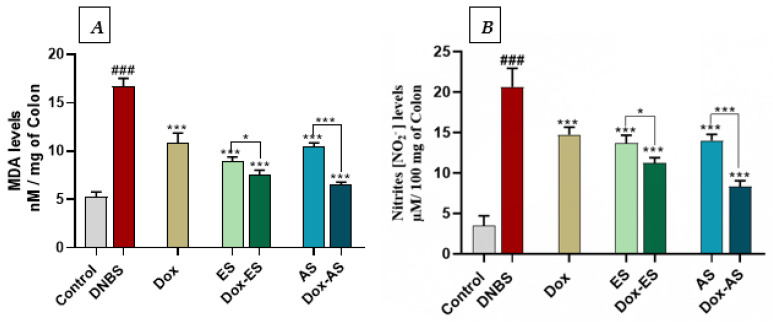
Effect of *A. platensis* extracts (ES and AS), alone or in combination with doxycycline (Dox), on colonic (**A**) malondialdehyde (MDA) levels and (**B**) nitrite (NO_2_^−^) levels production in DNBS-induced colitis in BALB/c mice. Data are expressed as mean ± standard deviation (*n* = 7 per group). Statistical analysis was performed using one-way ANOVA followed by Dunnett’s multiple comparisons test. *p* > 0.05, * *p* < 0.05, *** *p* < 0.001 vs. the DNBS group; **###** *p* < 0.001 vs. the control group.

To evaluate lipid peroxidation and nitrosative stress, MDA and nitrite levels were quantified in colonic tissues (Figure 6A,B). DNBS administration significantly increased both MDA (16.62 ± 0.96 nM/mg of colon) and NO levels (20.62 ± 2.31 µM/100 mg of colon) compared with the control group (5.23 ± 0.54 nM/mg of colon and 3.48 ± 1.21 µM/100 mg of colon, respectively; *p* < 0.001), confirming elevated oxidative stress in colitic mice. All treated groups, including doxycycline, ES, and AS monotherapies, as well as their combinations, showed a significant decrease in both markers compared to the DNBS group (*** *p* < 0.001). Specifically, MDA levels were reduced to 10.79 ± 1.09 nM/mg of colon (Dox), 8.90 ± 0.49 nM/mg of colon (ES), and 10.50 ± 0.36 nM/mg of colon (AS), while nitrite levels decreased to 14.73 ± 0.92, 14.37 ± 2.10, and 14.00 ± 0.76 µM/100 mg of colon, respectively. The combined treatments showed greater antioxidant effects. MDA and nitrite levels dropped to 7.50 ± 0.52 nM/mg of colon and 11.24 ± 0.65 µM/100 mg of colon in the Dox-ES group and to 6.57 ± 0.22 nM/mg of colon and 8.31 ± 0.72 µM/100 mg of colon in the Dox-AS group, respectively. A significant interaction between Dox and ES for both MDA (*p* < 0.0001) and NO (*p* = 0.0323), indicating a true synergistic effect, while the interaction between Dox and AS was significant for MDA (*p* = 0.0373) but not for NO (*p* = 0.8888), suggesting that the observed antioxidant effect of Dox-AS on MDA is synergistic, whereas its effect on NO is largely additive.

### 3.7. Antioxidant Defense Markers

**Figure 7:**
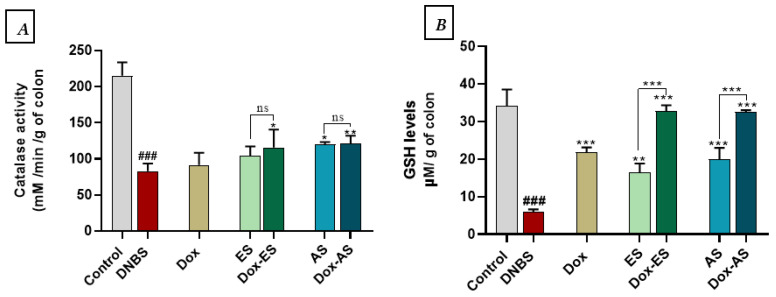
Effect of *A. platensis* extracts (ES and AS), alone or in combination with doxycycline (Dox), on (**A**) colonic catalase (CAT) activity and (**B**) reduced glutathione (GSH) levels in DNBS-induced colitis in BALB/c mice. Data are expressed as mean ± standard deviation (*n* = 7 per group). Statistical analysis was performed using one-way ANOVA followed by Dunnett’s multiple comparisons test. ns *p* > 0.05, * *p* < 0.05, ** *p* < 0.01 and *** *p* < 0.001 vs. the DNBS group; ### *p* < 0.001 vs. the control group.

To evaluate the antioxidant defense system in DNBS-induced colitis, CAT activity and GSH levels were quantified in colonic tissues (Figure 7A,B). DNBS administration led to a significant depletion of both CAT activity (82.93 ± 10.68 mM/min/g of colon) and GSH levels (5.91 ± 0.69 µM/g of colon) compared to the control group (215.10 ± 18.45 mM/min/g of colon and 34.16 ± 4.36 µM/g of colon, respectively; *p* < 0.001), indicating oxidative stress and impaired redox balance. Under monotherapy conditions, treatment with doxycycline (Dox) or the ethanolic extract (ES) led to a non-significant increase in CAT activity compared to the DNBS group (91.54 ± 16.88 and 104.22 ± 12.87 mM/min/g of colon, respectively; *p* > 0.05), while the aqueous extract (AS) induced a significant improvement (119.81 ± 3.42 mM/min/g of colon; *p* < 0.05). Regarding GSH levels, all monotherapies significantly restored glutathione content compared to DNBS (*p* < 0.001), with values of 21.84 ± 1.27 µM/g of colon (Dox), 16.38 ± 2.43 µM/g of colon (ES), and 19.92 ± 3.08 µM/g of colon (AS).

Combination therapies provided more substantial effects. Co-administration of Dox with ES or AS significantly enhanced CAT activity compared to the DNBS group (115.83 ± 24.66 and 119.81 ± 10.37 mM/min/g of colon, respectively), with *p* < 0.05 for Dox-ES and *p* < 0.01 for Dox-AS. No significant interaction between Dox and either extract for CAT (Dox-ES *p* = 0.8845, Dox-AS *p* = 0.5613) or GSH (Dox-ES *p* = 0.7827, Dox-AS *p* = 0.1047), indicating that the observed improvements are primarily driven by each treatment individually, without evidence of true synergy.

## 4. Discussion

This study introduces an innovative therapeutic strategy that combines doxycycline, an antibiotic with immunomodulatory properties, with ethanolic and aqueous extracts of *A. platensis* in a relapsing DNBS-induced colitis model in mice. The originality of this work lies in evaluating this combination during the reactivation phase of intestinal inflammation. This period closely mimics the relapse episodes characteristic of inflammatory bowel diseases such as Crohn’s disease and ulcerative colitis. To our knowledge, this is the first investigation to assess this synergistic approach in such a model, while also performing an in-depth GC–MS metabolomic characterization of the extracts after derivatization. This analysis revealed distinct bioactive profiles depending on the solvent used, with ES being rich in fatty acids and amino acids, whereas AS contained higher levels of polysaccharides and phytol. The combination of doxycycline with these extracts significantly improved clinical, histological, and biochemical parameters (MPO activity, MDA, GSH, NO levels, and CAT activity) compared to monotherapies, highlighting a genuine synergistic effect and offering a promising natural, integrative strategy for controlling IBD relapses.

A comprehensive understanding of the therapeutic potential of *A. platensis* requires a detailed exploration of its chemical composition, as the biological efficacy of natural extracts is inherently linked to their metabolite profiles. Accordingly, the initial focus of this investigation was placed on the metabolomic characterization of the ethanolic and aqueous fractions to establish mechanistic correlations between their molecular constituents and the biological responses observed in vivo.

The chemical characterization of *A. platensis* extracts (ES or AS) was performed by GC-MS following derivatization with MSTFA. This crucial step enhances the detection of polar and thermolabile metabolites such as those containing hydroxyl, carboxyl, and amine groups [43,44]. MSTFA replaces active hydrogens with trimethylsilyl groups, thereby increasing the volatility, thermal stability, and chromatographic behavior of these compounds, which otherwise would be difficult to analyze directly by GC-MS [45,46]. This derivatization strategy, widely validated in plant and microbial metabolomics, allows accurate profiling of amino acids, organic acids, sugars, and polyols, ensuring high sensitivity and reliability of the analysis [47,48]. The use of ethanol and water as solvents was chosen to maximize the extraction of a broad spectrum of bioactive compounds, with ethanol efficiently extracting non-polar to moderately polar molecules such as fatty acids and amino acids. In contrast, water targets highly polar compounds like polysaccharides and terpenoids [49,50,51]. This dual-solvent approach maximizes the diversity of extracted metabolites, producing chemically distinct yet biologically relevant extracts suitable for subsequent in vivo evaluations [49,52,53].

The GC-MS profiles revealed distinct chemical compositions between ES and AS extracts, reflecting the differential solubility and extraction capacity of the solvents used. The ES extract was dominated by a complex mixture of fatty acids and amino acids, with palmitic acid as the most abundant fatty acid, accompanied by significant levels of L-methionine and D-phenylalanine. These compounds are known for their multifaceted biological roles, including antioxidant activity and modulation of inflammatory pathways [28,54,55]. Methionine, in particular, is a key precursor in the synthesis of glutathione, a major intracellular antioxidant, while phenylalanine derivatives have been implicated in immune response regulation [56,57,58]. Additionally, the detection of 2-O-Glycerol-α-D-galactopyranoside suggests the presence of glycolipid-like structures, potentially contributing to membrane integrity and cellular signaling involved in anti-inflammatory processes [59,60,61,62]. The richness in these molecules provides a biochemical rationale for the observed therapeutic effects of the ES in mitigating colitis symptoms.

In contrast, AS extract was dominated by polysaccharides and terpenoid compounds, including D-Xylopyranose, α,α’-Trehalose, and phytol. The high abundance of sugars and sugar derivatives is consistent with the hydrophilic nature of the solvent, which efficiently extracts polar carbohydrates known for their immunomodulatory and prebiotic properties, key factors in gut health and inflammation control [63,64,65]. Phytol, a diterpenoid alcohol identified as a significant constituent, has demonstrated antioxidant and anti-inflammatory effects, further supporting the biological potential of the aqueous fraction [66,67,68,69,70]. The complementary chemical nature of both extracts offers a strong rationale for their synergistic therapeutic effects, particularly when combined with doxycycline, as their distinct bioactive constituents likely act on multiple convergent pathways to restore intestinal homeostasis.

In light of the multifactorial etiology of IBD, increasing attention is being paid to therapeutic approaches that simultaneously target immune dysregulation and gut microbiota imbalance [71,72]. Among these, antibiotics with anti-inflammatory potential, such as doxycycline, have shown promise due to their ability to modulate key inflammatory mediators (e.g., iNOS, TNF-α, IL-17) independently of their antimicrobial action [16,18,20]. However, the long-term use of such agents is constrained by the risk of adverse effects and microbiome disruption [73,74]. Therefore, combining such agents with safe, natural compounds capable of supporting gut homeostasis and exerting antioxidant, anti-inflammatory, and prebiotic effects appears as a promising alternative [13,25,75].

In this context, we propose the association of doxycycline with *A. platensis* extracts as a novel and effective therapeutic strategy to induce and maintain remission in IBD. The concentrations of both agents were selected based on prior studies and our own previous work to ensure optimal efficacy and safety in experimental colitis models [21,31,32]. To evaluate this hypothesis, a recurrent DNBS-induced colitis model in mice, mimicking the relapsing and chronic features of human IBD, was employed to assess the individual and combined efficacy of these treatments. Importantly, no cytotoxicity or adverse clinical effects were observed, confirming that the observed biochemical and histological outcomes accurately reflect the pharmacological effects of the treatments. Clinically, the progression of colitis, assessed through body weight evolution and the DAI, underscored the superior therapeutic performance of the combined treatment with doxycycline and *A. platensis* extracts. Although these two parameters are distinct, they converge in reflecting the severity of systemic and intestinal damage triggered by DNBS-induced inflammation [76,77]. The partial protection conferred by either agent alone indicates a moderate therapeutic effect; however, only the combined treatment produced a sustained attenuation of clinical symptoms during both the induction and relapse phases. This observation supports a synergistic mechanism, whereby the immunomodulatory actions of doxycycline are reinforced by the antioxidant, anti-inflammatory, and epithelial-protective properties of algal bioactives [18,34]. Supporting this, a previous study reported a similar combinatory approach in which doxycycline was co-administered with *Saccharomyces boulardii*, resulting in enhanced protection against colitis recurrence in the DSS-induced chronic model, reinforcing the relevance of dual-target therapies in chronic intestinal inflammation [21]. Furthermore, the use of minocycline, a tetracycline analogue, has been shown to alleviate colitis by mitigating body weight loss in chronic DSS models [21,35]. Similarly, both ethanolic and aqueous extracts of *A. platensis* have independently demonstrated the ability to alleviate clinical symptoms in models of acute and chronic colitis, reinforcing their therapeutic potential [28,31,32]. Thus, the improved clinical scores observed in the co-treated groups highlight the therapeutic relevance of this dual strategy for both remission induction and relapse prevention in experimental colitis.

The protective efficacy of *A. platensis* extracts against DNBS-induced colonic injury was further corroborated by macroscopic and histological analyses, which together provide a comprehensive view of tissue-level preservation and recovery. The colon weight-to-length ratio, widely recognized as a reliable macroscopic indicator of inflammation-induced edema and fibrosis, was significantly increased in untreated colitic mice, reflecting severe structural disruption [31,78]. This increase was only modestly attenuated by doxycycline monotherapy, consistent with its limited capacity to resolve established tissue remodeling. In contrast, both ES and AS extracts of *A. platensis* significantly reduced the ratio, indicating a notable anti-edematous effect and preservation of colonic structure. Notably, the combined treatments yielded even more pronounced improvements, with ratios approaching near-normal values, suggesting an additive therapeutic action. These results align with previous studies reporting that whole spirulina biomass or its derived extracts effectively reduced this inflammatory marker in both acute and chronic models of colitis, whether induced by DSS or DNBS [31,32]. Similarly, tetracyclines such as doxycycline and minocycline have demonstrated the capacity to attenuate this ratio in acute TNBS models, albeit to a lesser extent when used alone, thereby reinforcing the value of combined interventions targeting both inflammation and tissue damage [21].

This macroscopic preservation was further supported by microscopic observations, where histological assessment of the untreated colitic group revealed extensive mucosal damage, crypt distortion, and infiltration by polymorphonuclear and mononuclear cells, hallmark features of DNBS-induced epithelial injury. While doxycycline monotherapy offered limited structural recovery, treatments with either ES or AS conferred substantial histological improvement, notably by reducing submucosal edema, attenuating leukocyte infiltration, and partially restoring epithelial continuity. These findings are supported by previous investigations in which *A. platensis* extracts, whether aqueous, ethanolic, or methanolic, were shown to ameliorate histological scores in murine models of chemically induced colitis, through mechanisms involving antioxidant and anti-inflammatory pathways [28,31,32,79]. Importantly, the combination of *A. platensis* extracts with doxycycline resulted in near-complete normalization of tissue architecture, with preserved crypt structure and intact epithelium, closely resembling healthy controls. This pronounced effect further supports a synergistic interaction between the immunomodulatory properties of doxycycline and the multifaceted bioactivities of spirulina metabolites, including ROS scavenging, epithelial barrier restoration, and immune modulation [21,29,34,80]. Collectively, these macroscopic and microscopic findings substantiate the enhanced efficacy of the combined therapeutic strategy, which targets not only inflammation but also epithelial integrity, a central component in the pathophysiology and resolution of inflammatory bowel disease.

The assessment of colonic MPO activity provided valuable biochemical confirmation of the anti-inflammatory effects observed histologically. As a key enzyme released by activated neutrophils, MPO is widely recognized as a reliable marker of acute intestinal inflammation [81,82]. Its elevation in DNBS-induced colitis reflects not only neutrophilic infiltration but also the oxidative stress contributing to mucosal injury [82]. In this study, both *A. platensis* extracts and doxycycline were able to significantly reduce MPO activity, supporting their individual roles in dampening neutrophil-driven inflammation. Importantly, two-way ANOVA confirmed a true synergistic interaction for the Dox-ES combination, whereas the effect of Dox-AS appeared largely additive rather than synergistic. While the most pronounced reduction was observed in the combined treatment groups, particularly with Dox-ES, this synergistic effect likely stems from the complementary mechanisms of action of the two agents, with doxycycline exerting immunomodulatory effects, partly through inhibition of neutrophil chemotaxis and suppression of pro-oxidant enzymes, and *A. platensis* providing antioxidant and anti-inflammatory compounds capable of directly scavenging ROS and restoring epithelial integrity. Similar reductions in MPO activity have been reported in previous studies involving *Spirulina* extracts in models of TNBS and DSS-induced colitis [28,32]. Likewise, both doxycycline and minocycline have been shown to reduce MPO activity in TNBS-induced acute colitis, highlighting their capacity to limit leukocyte-mediated oxidative tissue injury [21]. Together, these results reinforce the therapeutic relevance of combining *A. platensis* with doxycycline to achieve a more efficient regulation of neutrophilic inflammation and mucosal protection in chronic intestinal disorders.

In line with the histological improvements, the modulation of oxidative and nitrosative stress appears to be a central mechanism involved in the beneficial effects of the treatments. DNBS-induced colitis led to a significant increase in MDA and nitrite levels, accompanied by a significant decrease in CAT activity and GSH content, indicating an imbalance in redox status [77,83,84,85]. Both *A. platensis* extracts and doxycycline, when administered alone, significantly reduced oxidative and nitrosative markers. The combination therapies exerted even stronger effects, suggesting a complementary mode of action. The two-way ANOVA analysis confirmed a true synergistic interaction for the Dox-ES combination on both MDA and NO, indicating a synergistic antioxidant effect for MDA and a synergistic anti-nitrosative effect for NO, whereas the effect of Dox-AS was synergistic for MDA but largely additive for NO. The antioxidant protection conferred by both extracts may be attributed to their specific GC-MS-identified constituents known for their radical-scavenging activity. In the ES extract, compounds such as L-Methionine, D-phenylalanine, and Palmitic acid have been reported to modulate oxidative stress responses [58,86]. Similarly, the AS extract, enriched in D-Xylopyranose, Phytol and α,α’-Trehalose, may contribute to cellular redox balance through multiple mechanisms, including membrane stabilization and the quenching of reactive species [69,87,88,89]. Doxycycline contributed by limiting neutrophil activation and reducing iNOS expression, consistent with previous findings demonstrating its ability to decrease nitrite production in LPS-stimulated RAW 264.7 macrophages and in experimental colitis models (DSS and TNBS), including in association with probiotics [21]. Although the combinations did not significantly enhance the increase in CAT activity compared to monotherapies, the more substantial restoration of GSH levels indicates a favorable interaction between the two treatments. However, no significant synergistic interaction was detected for CAT or GSH, indicating that these improvements are primarily driven by each treatment individually. This dual mechanism, by reducing ROS production and supporting antioxidant defenses, shows the benefit of combining anti-inflammatory and antioxidant strategies in treating colitis.

The therapeutic effects observed in this study can be attributed to the bioactive composition and the specific mechanistic actions of each extract. ES extract, rich in methionine and tyramine, likely exerted anti-inflammatory effects by downregulating key mediators such as TNF-α, IL-6, and COX-2, while enhancing IL-4 production, thus reducing the immune response toward a regulatory profile [90,91]. On the other hand, AS extract, characterized by a high content of phytol and polysaccharides, demonstrated potent antioxidant and anti-inflammatory activities, as supported by its ability to scavenge free radicals, suppress iNOS and PGE2 expression, and promote mucosal repair [92,93]. These effects are consistent with previous reports showing that *A. platensis* mitigates experimental colitis by modulating both oxidative and inflammatory pathways [31,32,79,94]. Additionally, doxycycline has been shown to modulate multiple molecular targets relevant to IBD pathogenesis, including Th1/Th17 cytokines (IL-12, IL-1β, IL-17, TNF-α), cell adhesion molecules (ICAM-1), barrier integrity proteins (MUC-1, MUC-3), and enzymes such as MMPs and iNOS [21]. The combination of doxycycline with *A. platensis* extracts appears to leverage their respective strengths, doxycycline primarily reducing leukocyte activation and enzymatic tissue degradation, while the extracts enhance antioxidant defense and regulate cytokine signaling, resulting in a synergistic interaction that effectively attenuates inflammation and oxidative stress at both cellular and molecular levels.

## 5. Conclusions

This study underscores the anticolitic effects of combining *A. platensis* extracts with doxycycline as a dual-target approach to manage intestinal inflammation. Through integrated chemical profiling and a well-established recurrent colitis model, both ethanolic and aqueous extracts exhibited significant protection against colitis-induced tissue damage. Their combination with doxycycline significantly enhanced clinical, histological, and biochemical outcomes in a relapsing colitis setting, pointing to a synergistic interaction that improves therapeutic efficacy across both acute and relapsing phases of the disease. While the findings provide strong preclinical evidence for this combinatory approach, further investigations are needed to delineate the precise molecular pathways involved and to validate the translational relevance of this strategy in clinical contexts.

## Data Availability

The original contributions presented in this study are included in the article/Supplementary Material. Further inquiries can be directed to the corresponding author.

## Supplementary Materials

The following supporting information can be downloaded at: https://www.mdpi.com/article/10.3390/jox15050160/s1, Figure S1: GC-MS chromatogram of the derivatized *A. platensis* ethanolic extract (ES); Table S1: Chemical profile of the derivatized *A. platensis* ethanolic extract (ES) identified by GC–MS; Figure S2: GC-MS chromatogram of the derivatized *A. platensis* aqueous extract (AS); Table S2: Chemical profile of the derivatized *A. platensis* aqueous extract (AS) identified by GC–MS; Table S3: Original uncropped histological sections.

## Abbreviations

CD	Crohn’s Disease
COX-2	Cyclooxygenase-2
DAI	Disease Activity Index
DNBS	Dinitrobenzene Sulfonic Acid
GSH	Reduced Glutathion
HTAB	Hexadecyl-Trimethyl-Ammonium Bromide
IBD	Inflammatory Bowel Disease
ICAM-3	Intercellular Adhesion Molecule-1
IL-6	Interleukin 6.
iNOS	Inducible Nitric Oxide Synthase
MDA	Malondialdehyde.
MPO	Myeloperoxydase.
MUC-1	Mucin-1
MUC-3	Mucin-3
NF-κB	Nuclear factor kappa B
NO	Nitric Oxide.
PGE2	Prostaglandin E2.
PMS	Post-Mitochondrial Supernatant.
RNS	Reactive Nitrogen Species.
ROS	Reactive Oxygen Species.
TNF-α	Tumor Necrosis Factor-Alpha.
UC	Ulcerative Colitis.

## References

[1] Calvez V., Puca P., Di Vincenzo F., Del Gaudio A., Bartocci B., Murgiano M., Iaccarino J., Parand E., Napolitano D., Pugliese D. (2025). Novel Insights into the Pathogenesis of Inflammatory Bowel Diseases. Biomedicines.

[2] Kong L., Chen S., Huang S., Zheng A., Gao S., Ye J., Hua C. (2024). Challenges and Opportunities in Inflammatory Bowel Disease: From Current Therapeutic Strategies to Organoid-Based Models. Inflamm. Res..

[3] Bribi N., Yanat B., Ouahmed-Boudaoud H. (2019). Immunopathogenesis of Ulcerative Colitis and Crohn’s Disease. Int. J. Adv. Res. Microbiol. Immunol..

[4] Alemany-Cosme E., Sáez-González E., Moret I., Mateos B., Iborra M., Nos P., Sandoval J., Beltrán B. (2021). Oxidative Stress in the Pathogenesis of Crohn’s Disease and the Interconnection with Immunological Response, Microbiota, External Environmental Factors, and Epigenetics. Antioxidants.

[5] Megha K.B., Joseph X., Akhil V., Mohanan P.V. (2021). Cascade of Immune Mechanism and Consequences of Inflammatory Disorders. Phytomedicine.

[6] Park J., Cheon J.H. (2022). Updates on Conventional Therapies for Inflammatory Bowel Diseases: 5-Aminosalicylates, Corticosteroids, Immunomodulators, and Anti-TNF-α. Korean J. Intern. Med..

[7] Sebastian S.A., Kaiwan O., Co E.L., Mehendale M., Mohan B.P. (2024). Current Pharmacologic Options and Emerging Therapeutic Approaches for the Management of Ulcerative Colitis: A Narrative Review. Spartan Med. Res. J..

[8] Subramanian R., Triadafilopoulos G. (2016). Care of Inflammatory Bowel Disease Patients in Remission. Gastroenterol. Rep..

[9] Cai Z., Wang S., Li J. (2021). Treatment of Inflammatory Bowel Disease: A Comprehensive Review. Front. Med..

[10] Bourgonje A.R., Feelisch M., Faber K.N., Pasch A., Dijkstra G., van Goor H. (2020). Oxidative Stress and Redox-Modulating Therapeutics in Inflammatory Bowel Disease. Trends Mol. Med..

[11] Tian T., Wang Z., Zhang J. (2017). Pathomechanisms of Oxidative Stress in Inflammatory Bowel Disease and Potential Antioxidant Therapies. Oxidative Med. Cell. Longev..

[12] He B., Lu C., Zheng G., He X., Wang M., Chen G., Zhang G., Lu A. (2016). Combination Therapeutics in Complex Diseases. J. Cell. Mol. Med..

[13] Malik M.A., Wani M.Y., Hashmi A.A., Wani M.Y., Ahmad A. (2020). Chapter 1—Combination Therapy: Current Status and Future Perspectives. Combination Therapy Against Multidrug Resistance.

[14] Sagar J. (2010). Doxycycline in Clinical Medicine. Clin. Med. Insights Ther..

[15] Majewski M. (2014). A Current Opinion on the Safety and Efficacy of Doxycycline Including Parenteral Administration—A Review. Pol. Ann. Med..

[16] Navarro-Triviño F.J., Pérez-López I., Ruiz-Villaverde R. (2020). Doxycycline, an Antibiotic or an Anti-Inflammatory Agent? The Most Common Uses in Dermatology. Actas Dermosifiliogr..

[17] Clemens D.L., Duryee M.J., Sarmiento C., Chiou A., McGowan J.D., Hunter C.D., Schlichte S.L., Tian J., Klassen L.W., O’Dell J.R. (2018). Novel Antioxidant Properties of Doxycycline. Int. J. Mol. Sci..

[18] Di Caprio R., Lembo S., Di Costanzo L., Balato A., Monfrecola G. (2015). Anti-Inflammatory Properties of Low and High Doxycycline Doses: An In Vitro Study. Mediators Inflamm..

[19] Nakagawa T., Kakizoe Y., Iwata Y., Miyasato Y., Mizumoto T., Adachi M., Izumi Y., Kuwabara T., Suenaga N., Narita Y. (2018). Doxycycline Attenuates Cisplatin-Induced Acute Kidney Injury through Pleiotropic Effects. Am. J. Physiol. Ren. Physiol..

[20] Ledder O. (2019). Antibiotics in Inflammatory Bowel Diseases: Do We Know What We’re Doing?. Transl. Pediatr..

[21] Garrido-Mesa J., Algieri F., Rodriguez-Nogales A., Utrilla M.P., Rodriguez-Cabezas M.E., Zarzuelo A., Ocete M.A., Garrido-Mesa N., Galvez J. (2015). A New Therapeutic Association to Manage Relapsing Experimental Colitis: Doxycycline plus *Saccharomyces boulardii*. Pharmacol. Res..

[22] Garrido-Mesa J., Rodríguez-Nogales A., Algieri F., Vezza T., Hidalgo-Garcia L., Garrido-Barros M., Utrilla M.P., Garcia F., Chueca N., Rodriguez-Cabezas M.E. (2018). Immunomodulatory Tetracyclines Shape the Intestinal Inflammatory Response Inducing Mucosal Healing and Resolution. Br. J. Pharmacol..

[23] Chan P.A., Le Brazidec D.L., Becasen J.S., Martin H., Kapadia J., Reno H., Bachmann L., Barbee L.A. (2023). Safety of Longer-Term Doxycycline Use: A Systematic Review and Meta-Analysis with Implications for Bacterial STI Chemoprophylaxis. Sex. Transm. Dis..

[24] Carpenter L., Miller S., Flynn E., Choo J.M., Collins J., Shoubridge A.P., Gordon D., Lynn D.J., Whitehead C., Leong L.E.X. (2024). Exposure to Doxycycline Increases Risk of Carrying a Broad Range of Enteric Antimicrobial Resistance Determinants in an Elderly Cohort. J. Infect..

[25] Aware C.B., Patil D.N., Suryawanshi S.S., Mali P.R., Rane M.R., Gurav R.G., Jadhav J.P. (2022). Natural Bioactive Products as Promising Therapeutics: A Review of Natural Product-Based Drug Development. S. Afr. J. Bot..

[26] Saharan V., Jood S. (2017). NUTRITIONAL COMPOSITION OF SPIRULINA PLATENSIS POWDER AND ITS ACCEPTABILITY IN FOOD PRODUCTS. Int. J. Adv. Res..

[27] Seghiri R., Kharbach M., Essamri A. (2019). Functional Composition, Nutritional Properties, and Biological Activities of Moroccan *Spirulina* Microalga. J. Food Qual..

[28] Morsy M.A., Gupta S., Nair A.B., Venugopala K.N., Greish K., El-Daly M. (2019). Protective Effect of Spirulina Platensis Extract against Dextran-Sulfate-Sodium-Induced Ulcerative Colitis in Rats. Nutrients.

[29] Agustina S., Aidha N.N., Oktarina E., Kurniati N.F. (2021). Evaluation of Antioxidant and Wound Healing Activities of *Spirulina* sp. Extract. Egypt. J. Chem..

[30] Abd El-Fattah Elshouny W., Mohammed El-Sheekh M., Zaki Sabae S., Abdelfattah Khalil M., Mohamed Badr H. (2017). ANTIMICROBIAL ACTIVITY of SPIRULINA PLATENSIS AGAINST AQUATIC BACTERIAL ISOLATES. J. Microbiol. Biotechnol. Food Sci..

[31] Aziez M., Bribi N., Sofiane M.M., Riad F., Affenai S. (2024). Intestinal Anti-Inflammatory and Antioxidant Potential of Arthrospira Platensis Aqueous Extract on DNBS-Induced Colitis in BALB/c Mice. Curr. Chem. Biol..

[32] Aziez M., Suharoschi R., Merakeb M.S., Pop O.L., Ciont C., Ranga F., Ferhat R., Affenai S., Vodnar D.C., Cozma A. (2025). Phenolic Profiling and Bioactive Properties of Arthrospira Platensis Extract in Alleviating Acute and Sub-Chronic Colitis. Int. J. Mol. Sci..

[33] Hutadilok-Towatana N., Reanmongkol W., Panichayupakaranant P. (2010). Evaluation of the Toxicity of Arthrospira (Spirulina) Platensis Extract. J. Appl. Phycol..

[34] Khafaga A.F., El-Sayed Y.S. (2018). Spirulina Ameliorates Methotrexate Hepatotoxicity via Antioxidant, Immune Stimulation, and Proinflammatory Cytokines and Apoptotic Proteins Modulation. Life Sci..

[35] Garrido-Mesa N., Camuesco D., Arribas B., Comalada M., Bailón E., Cueto-Sola M., Utrilla P., Nieto A., Zarzuelo A., Rodríguez-Cabezas M.E. (2011). The Intestinal Anti-Inflammatory Effect of Minocycline in Experimental Colitis Involves Both Its Immunomodulatory and Antimicrobial Properties. Pharmacol. Res..

[36] Khajah M.A., Hawai S., Barakat A., Albaloushi A., Alkharji M., Masocha W. (2023). Minocycline Synergizes with Corticosteroids in Reducing Colitis Severity in Mice via the Modulation of Pro-Inflammatory Molecules. Front. Pharmacol..

[37] Garcia F.A.d.O., Sales-Campos H., Yuen V.G., Machado J.R., Viana G.S.d.B., McNeill J.H. (2020). *Arthrospira* (*Spirulina*) *Platensis* Attenuates Dextran Sulfate Sodium-Induced Colitis in Mice by Suppressing Key Pro-Inflammatory Cytokines. Korean J. Gastroenterol..

[38] Hove T.T., Corbaz A., Amitai H., Aloni S., Belzer I., Graber P., Drillenburg P., Van Deventer S.J.H., Chvatchko Y., Te Velde A.A. (2001). Blockade of Endogenous IL-18 Ameliorates TNBS-Induced Colitis by Decreasing Local TNF-α Production in Mice. Gastroenterology.

[39] Merakeb M.S., Bribi N., Ferhat R., Aziez M., Yanat B. (2023). Alkaloids Extract from Linum Usitatissimum Attenuates 12-OTetradecanoylphorbol- 13-Acetate (TPA)-Induced Inflammation and Oxidative Stress in Mouse Skin. Anti-Inflamm. Anti-Allergy Agents Med. Chem..

[40] Rehman I.U., Saleem M., Raza S.A., Bashir S., Muhammad T., Asghar S., Qamar M.U., Shah T.A., Bin Jardan Y.A., Mekonnen A.B. (2024). Anti-Ulcerative Colitis Effects of Chemically Characterized Extracts from Calliandra Haematocephala in Acetic Acid-Induced Ulcerative Colitis. Front. Chem..

[41] Avula S.K., Khan A., Halim S.A., Rehman N.U., Karim N., Khan I., Csuk R., Das B., Al-Harrasi A. (2022). Synthesis and Antidepressant-like Effects of New *5-Epi*-Incensole *and 5-Epi*—Incensole Acetate in Chronic Unpredictable Mild Stress Model of Depression; Behavioural and Biochemical Correlates. Biomed. Pharmacother..

[42] Rathore P., Arora I., Rastogi S., Akhtar M., Singh S., Samim M. (2020). Collagen Nanoparticle-Mediated Brain Silymarin Delivery: An Approach for Treating Cerebral Ischemia and Reperfusion-Induced Brain Injury. Front. Neurosci..

[43] Zhao S., Li L. (2021). Chemical Derivatization for Polar Metabolome Analysis. MALDI Mass Spectrometry Imaging.

[44] Huang T., Toro M., Lee R., Hui D.S., Edwards J.L. (2018). Multi-Functional Derivatization of Amine, Hydroxyl, and Carboxylate Groups for Metabolomic Investigations of Human Tissue by Electrospray Ionization Mass Spectrometry. Analyst.

[45] Zhou Y.-Q., Wang Z.-J., Jia N. (2007). Formation of Multiple Trimethylsilyl Derivatives in the Derivatization of 17alpha-Ethinylestradiol with BSTFA or MSTFA Followed by Gas Chromatography-Mass Spectrometry Determination. J. Environ. Sci. China.

[46] Parvatam R., Singh R., Sharma R. (2023). Comparison of Different Derivatising Reagents in Identification of Milk Metabolites Using GC–MS. Int. Dairy J..

[47] Liao H.-Y., Wang C.-Y., Lee C.-H., Kao H.-L., Wu W.-K., Kuo C.-H. (2021). Development of an Efficient and Sensitive Chemical Derivatization-Based LC-MS/MS Method for Quantifying Gut Microbiota-Derived Metabolites in Human Plasma and Its Application in Studying Cardiovascular Disease. J. Proteome Res..

[48] Xue G., Su S., Yan P., Shang J., Wang J., Yan C., Li J., Wang Q., Xiong X., Xu H. (2022). Integrative Analyses of Widely Targeted Metabolomic Profiling and Derivatization-Based LC-MS/MS Reveals Metabolic Changes of *Zingiberis Rhizoma* and Its Processed Products. Food Chem..

[49] Plaskova A., Mlcek J. (2023). New Insights of the Application of Water or Ethanol-Water Plant Extract Rich in Active Compounds in Food. Front. Nutr..

[50] Monroy Y.M., Rodrigues R.A.F., Sartoratto A., Cabral F.A. (2016). Influence of Ethanol, Water, and Their Mixtures as Co-Solvents of the Supercritical Carbon Dioxide in the Extraction of Phenolics from Purple Corn Cob (*Zea mays* L.). J. Supercrit. Fluids.

[51] Jha A.K., Sit N. (2022). Extraction of Bioactive Compounds from Plant Materials Using Combination of Various Novel Methods: A Review. Trends Food Sci. Technol..

[52] Tuhanioglu A., Kaur S., De Barros G.L., Ahmadzadeh S., Threlfall R., Ubeyitogullari A. (2025). Optimizing Ethanol-Water Cosolvent Systems for Green Supercritical Carbon Dioxide Extraction of Muscadine Grape Pomace Polyphenols. ACS Omega.

[53] Lezoul N.E.H., Belkadi M., Habibi F., Guillén F. (2020). Extraction Processes with Several Solvents on Total Bioactive Compounds in Different Organs of Three Medicinal Plants. Molecules.

[54] Mühling M., Belay A., Whitton B.A. (2005). Variation in Fatty Acid Composition of Arthrospira (Spirulina) Strains. J. Appl. Phycol..

[55] Taiti C., Di Vito M., Di Mercurio M., Costantini L., Merendino N., Sanguinetti M., Bugli F., Garzoli S. (2024). Detection of Secondary Metabolites, Proximate Composition and Bioactivity of Organic Dried Spirulina (*Arthrospira platensis*). Appl. Sci..

[56] Ji J., Xu Y., Zheng M., Luo C., Lei H., Qu H., Shu D. (2019). Methionine Attenuates Lipopolysaccharide-Induced Inflammatory Responses via DNA Methylation in Macrophages. ACS Omega.

[57] Lauinger L., Kaiser P. (2021). Sensing and Signaling of Methionine Metabolism. Metabolites.

[58] Levine R.L., Moskovitz J., Stadtman E.R. (2000). Oxidation of Methionine in Proteins: Roles in Antioxidant Defense and Cellular Regulation. IUBMB Life.

[59] Hosseini A., Mas J. (2021). The β-Galactosidase Assay in Perspective: Critical Thoughts for Biosensor Development. Anal. Biochem..

[60] Sharma S.K., Sharma S.P., Leblanc R.M. (2021). Methods of Detection of β-Galactosidase Enzyme in Living Cells. Enzyme Microb. Technol..

[61] Kremer B.P., Kirst G.O. (1981). Biosynthesis of 2-*O*-D-Glycerol-*α*-D-Galactopyranoside (Floridoside) in Marine *Rhodophyceae*. Plant Sci. Lett..

[62] Liu D., Wang Y., Lu Z., Lv F., Bie X., Zhao H. (2023). Separation, Characterization and Anti-Inflammatory Activities of Galactoglycerolipids from *Perilla frutescens* (L.) Britton. Nat. Prod. Res..

[63] Jiang Y., Wei Y., Zhou Q.-Y., Sun G.-Q., Fu X.-P., Levin N., Zhang Y., Liu W.-Q., Song N., Mohammed S. (2024). Direct Radical Functionalization of Native Sugars. Nature.

[64] Li S., Lv M., Zhang S., Xu H. (2021). Advances on Monosaccharides and Oligosaccharides: Structural Modifications and Bioactivities. Mini Rev. Med. Chem..

[65] Arnone D., Chabot C., Heba A.-C., Kökten T., Caron B., Hansmannel F., Dreumont N., Ananthakrishnan A.N., Quilliot D., Peyrin-Biroulet L. (2022). Sugars and Gastrointestinal Health. Clin. Gastroenterol. Hepatol..

[66] Araújo R.P.N., da Silva Freitas F.V., Nunes D.B., da Silva Brito A.K., da Costa D.S., de Sousa D.P., de Cássia Meneses Oliveira R., dos Santos R.F. (2024). Investigating the Pharmacological Potential of Phytol on Experimental Models of Gastric Ulcer in Rats. Naunyn. Schmiedebergs Arch. Pharmacol..

[67] Olofsson P., Hultqvist M., Hellgren L.I., Holmdahl R., Jacob C., Kirsch G., Slusarenko A., Winyard P.G., Burkholz T. (2014). Phytol: A Chlorophyll Component with Anti-Inflammatory and Metabolic Properties. Recent Advances in Redox Active Plant and Microbial Products: From Basic Chemistry to Widespread Applications in Medicine and Agriculture.

[68] Pejin B., Savic A., Sokovic M., Glamoclija J., Ciric A., Nikolic M., Radotic K., Mojovic M. (2014). Further in Vitro Evaluation of Antiradical and Antimicrobial Activities of Phytol. Nat. Prod. Res..

[69] Silva R.O., Sousa F.B.M., Damasceno S.R.B., Carvalho N.S., Silva V.G., Oliveira F.R.M.A., Sousa D.P., Aragão K.S., Barbosa A.L.R., Freitas R.M. (2014). Phytol, a Diterpene Alcohol, Inhibits the Inflammatory Response by Reducing Cytokine Production and Oxidative Stress. Fundam. Clin. Pharmacol..

[70] Sadukha S., Bhayani A., Padariya H., Vaghela P., Mishra S., Ghosh A., Dineshkumar R. (2023). Performance Evaluation of Microalgal Strains for Concurrent Production of High-Value Bio-Actives Lutein and Phytol: A Step Forward towards the Multi-Product Paradigm. Biocatal. Agric. Biotechnol..

[71] Garavaglia B., Vallino L., Amoruso A., Pane M., Ferraresi A., Isidoro C. (2024). The Role of Gut Microbiota, Immune System, and Autophagy in the Pathogenesis of Inflammatory Bowel Disease: Molecular Mechanisms and Therapeutic Approaches. Asp. Mol. Med..

[72] Ko J.K., Auyeung K.K. (2014). Inflammatory Bowel Disease: Etiology, Pathogenesis and Current Therapy. Curr. Pharm. Des..

[73] Eljaaly K., Alghamdi H., Almehmadi H., Aljawi F., Hassan A., Thabit A. (2023). Long-Term Gastrointestinal Adverse Effects of Doxycycline. J. Infect. Dev. Ctries..

[74] Wang J., Gan Q., Zhang H., Xing Q., Wu Y., Su X., Zhang N., Wu K. (2023). Long-Term Doxycycline Therapy for Stable Chronic Obstructive Pulmonary Disease: Do the Benefits Outweigh the Drawbacks?. Am. J. Respir. Crit. Care Med..

[75] Duan L., Cheng S., Li L., Liu Y., Wang D., Liu G. (2021). Natural Anti-Inflammatory Compounds as Drug Candidates for Inflammatory Bowel Disease. Front. Pharmacol..

[76] Park Y.H., Kim N., Shim Y.K., Choi Y.J., Nam R.H., Choi Y.J., Ham M.H., Suh J.H., Lee S.M., Lee C.M. (2015). Adequate Dextran Sodium Sulfate-Induced Colitis Model in Mice and Effective Outcome Measurement Method. J. Cancer Prev..

[77] Morampudi V., Bhinder G., Wu X., Dai C., Sham H.P., Vallance B.A., Jacobson K. (2014). DNBS/TNBS Colitis Models: Providing Insights into Inflammatory Bowel Disease and Effects of Dietary Fat. J. Vis. Exp. JoVE.

[78] Ahn S.-I., Cho S., Choi N.-J. (2020). Effect of Dietary Probiotics on Colon Length in an Inflammatory Bowel Disease-Induced Murine Model: A Meta-Analysis. J. Dairy Sci..

[79] Abdel-Daim M.M., Farouk S.M., Madkour F.F., Azab S.S. (2015). Anti-Inflammatory and Immunomodulatory Effects of *Spirulina Platensis* in Comparison to *Dunaliella salina* in Acetic Acid-Induced Rat Experimental Colitis. Immunopharmacol. Immunotoxicol..

[80] Wu Q., Liu L., Miron A., Klímová B., Wan D., Kuča K. (2016). The Antioxidant, Immunomodulatory, and Anti-Inflammatory Activities of Spirulina: An Overview. Arch. Toxicol..

[81] Hanning N., De Man J.G., De Winter B.Y. (2023). Measuring Myeloperoxidase Activity as a Marker of Inflammation in Gut Tissue Samples of Mice and Rat. Bio-Protocol..

[82] Mancini S., Mariani F., Sena P., Benincasa M., Roncucci L. (2017). Myeloperoxidase Expression in Human Colonic Mucosa Is Related to Systemic Oxidative Balance in Healthy Subjects. Redox Rep. Commun. Free Radic. Res..

[83] da Silva V.C., de Araújo A.A., Araújo D.F.d.S., Lima M.C.J.S., Vasconcelos R.C., de Araújo Júnior R.F., Langasnner S.M.Z., Pedrosa M. (2018). de F.F.; de Medeiros, C.A.C.X.; Guerra, G.C.B. Intestinal Anti-Inflammatory Activity of the Aqueous Extract from Ipomoea Asarifolia in DNBS-Induced Colitis in Rats. Int. J. Mol. Sci..

[84] Bribi N., Algieri F., Nogales A., Vezza T., Garrido-Mesa J., Utrilla M., Contreras M.d.M., Maiza F., Segura Carretero A., Rodriguez-Cabezas M. (2016). Intestinal Anti-Inflammatory Effects of Total Alkaloid Extract from Fumaria Capreolata in the DNBS Model of Mice Colitis and Intestinal Epithelial CMT93 Cells. Phytomedicine.

[85] Eissa N., Kermarrec L., Hussein H., Bernstein C.N., Ghia J.-E. (2017). Appropriateness of Reference Genes for Normalizing Messenger RNA in Mouse 2,4-Dinitrobenzene Sulfonic Acid (DNBS)-Induced Colitis Using Quantitative Real Time PCR. Sci. Rep..

[86] Wang X., Zhang C., Bao N. (2023). Molecular Mechanism of Palmitic Acid and Its Derivatives in Tumor Progression. Front. Oncol..

[87] Mizunoe Y., Kobayashi M., Sudo Y., Watanabe S., Yasukawa H., Natori D., Hoshino A., Negishi A., Okita N., Komatsu M. (2018). Trehalose Protects against Oxidative Stress by Regulating the Keap1-Nrf2 and Autophagy Pathways. Redox Biol..

[88] Yang L., Zhao X., Zhu H., Paul M., Zu Y., Tang Z. (2014). Exogenous Trehalose Largely Alleviates Ionic Unbalance, ROS Burst, and PCD Occurrence Induced by High Salinity in Arabidopsis Seedlings. Front. Plant Sci..

[89] Kiruthiga C., Balan D.J., Jafni S., Anandan D.P., Devi K.P. (2024). Phytol and (−)-α-Bisabolol Synergistically Trigger Intrinsic Apoptosis through Redox and Ca2+ Imbalance in Non-Small Cell Lung Cancer. Biocatal. Agric. Biotechnol..

[90] Babusyte A., Kotthoff M., Fiedler J., Krautwurst D. (2013). Biogenic Amines Activate Blood Leukocytes via Trace Amine-Associated Receptors TAAR1 and TAAR2. J. Leukoc. Biol..

[91] Patel A., Thompson A., Abdelmalek L., Adams-Huet B., Jialal I. (2019). The Relationship between Tyramine Levels and Inflammation in Metabolic Syndrome. Horm. Mol. Biol. Clin. Investig..

[92] Yongkhamcha B., Buddhakala N. (2023). Phytochemical Compositions, Nutritional Contents, Cytotoxicity and Anti-Inflammatory Activity of Different Extracts from Spirogyra Neglecta (Hassall) Kützing. Trends Sci..

[93] Aba P.E., Odugbo I.O. (2024). Metabolite Profiling and in Silico Studies to Elucidate the Anti-Inflammatory Properties of Pterocarpus Milbreadii. Curr. Enzyme Inhib..

[94] Coskun Z.K., Kerem M., Gurbuz N., Omeroglu S., Pasaoglu H., Demirtas C., Lortlar N., Salman B., Pasaoglu O.T., Turgut H.B. (2011). The Study of Biochemical and Histopathological Effects of Spirulina in Rats with TNBS-Induced Colitis. Bratisl. Lek. Listy.

