# Supplementary material for: A Synergistic Approach with Doxycycline and Spirulina Extracts in DNBS-Induced Colitis: Enhancing Remission and Controlling Relapse
Source: J Xenobiot. 2025 Oct 3;15(5):160. doi: 10.3390/jox15050160 (PMC12565093; doi:10.3390/jox15050160)
Supplement: Supplementary file 1 [file jox-15-00160-s001.zip › jox-3748073-supplementary.pdf]

A Synergistic Approach with Doxycycline and Spirulina Extracts in DNBS-Induced Colitis: Enhancing Remission and Controlling Relapse

Meriem Aziez [1], Mohamed Malik Mahdjoub [2,3], Tahar Benayad [4], Ferroudja Abbas [4], Sarah Hamid [1], Hamza Moussa [3], Ibrahima Mamadou Sall [5], Hichem Tahraoui [6,7], Abdeltif Amrane [7*] and Noureddine Bribi [1]

[1] Laboratoire de Biotechnologie Végétale et Ethnobotanique, Faculté des Sciences de la Nature et de la Vie, Université de Bejaia, Bejaia 06000, Algeria; meriem.aziez@univ-bejaia.dz (M.A.); sarah.hamid@univ-bejaia.dz (S.H.); noureddine.bribi@univ-bejaia.dz (N.B.)
[2] Département des Sciences de la Nature et de la Vie, Faculté des Sciences, Université d'Alger 1, Didouche Mourad, Alger 16000, Algeria; m.mahdjoub@univ-bouira.dz (M.M.M.)
[3] Département des Sciences Biologiques, Faculté des Sciences de la Nature et de la Vie et des Sciences de la Terre, Université de Bouira, Bouira 10000, Algeria; h.moussa@univ-bouira.dz (H.M.)
[4] Laboratoire Central de la Police Scientifique, Alger 16000, Algeria; taharbenayad1@gmail.com (T.B.); ferouab@yahoo.fr (F.A.)
[5] Department of Anatomic Pathology, Faculty of Veterinary Medicine, University of Agricultural Sciences and Veterinary Medicine of Cluj-Napoca, 400372 Cluj-Napoca, Romania; ibrahima.sall@student.usamvcluj.ro
[6] Laboratory of Biomaterials and Transport Phenomena, University of Medea, Medea 26000, Algeria; hichemm.tahraouii@gmail.com
[7] Ecole Nationale Supérieure de Chimie de Rennes, Université de Rennes, CNRS, ISCR—UMR6226, F-35000 Rennes, France
* Correspondence: abdeltif.amrane@univ-rennes.fr

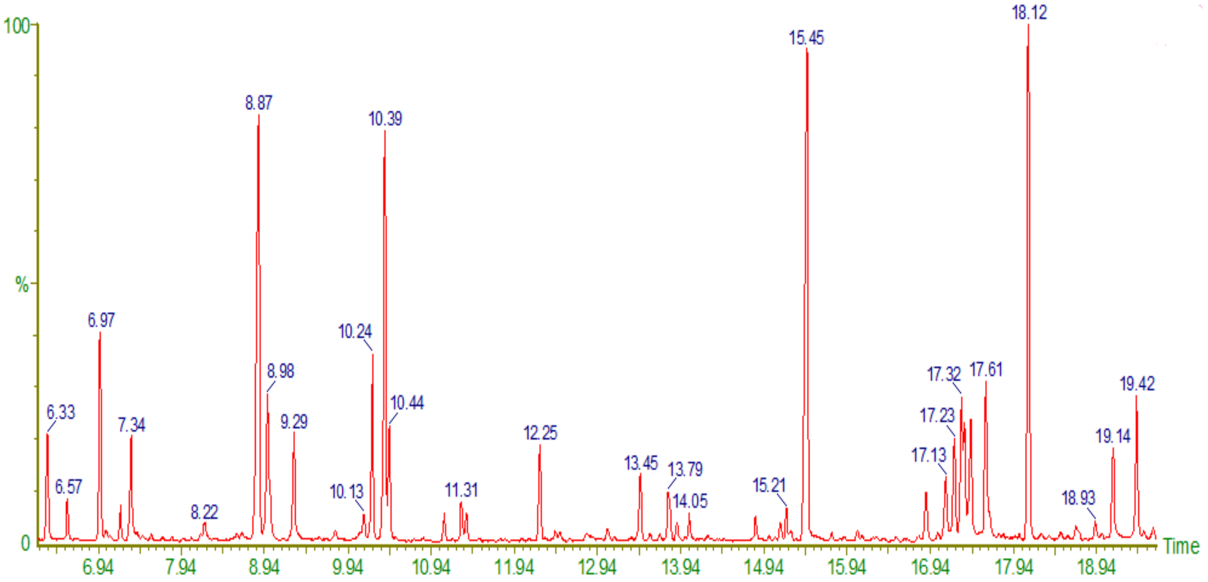

Figure S1. GC-MS chromatogram of the derivatized *A. platensis* ethanolic extract (ES).

Table S1. Chemical profile of the derivatized *A. platensis* ethanolic extract (ES) identified by GC–MS.

Peak	RT (min)	Compounds	Match Score (%)	Molecular Weight (Da)	TMS Derivative	Molecular formula	Area %
**1**	6.33	Uracil	90.2	256	2 TMS	$C_{10}H_{20}O_2N_2Si_2$	**-**
**2**	6.97	L-Threonine	96.7	335	3 TMS	$C_{13}H_{33}NO_3Si_3$	**5.66**
**3**	7.34	L-Methionine	97.4	221	1 TMS	$C_8H_{19}NO_2SSi$	**3.21**
**4**	8.87	L-Methionine	97.2	293	2 TMS	$C_{11}H_{27}NO_2SSi_2$	**13.62**
**5**	9.29	DL-Phenylalanine	96.5	237	1 TMS	$C_{12}H_{19}NO_2Si$	**3.62**
**6**	10.24	L-Glutamic acid.	95.3	363	3 TMS	$C_{14}H_{33}NO_4Si_3$	**4.76**
**7**	10.39	D-phenylalanine	96.4	309	2 TMS	$C_{15}H_{27}NO_2Si_2$	**11.79**
**8**	10.44	Tyramine	95.3	281	2 TMS	$C_{14}H_{27}NOSi_2$	**2.35**
**9**	12.25	Glycerophosphoric acid	91.8	460	4 TMS	$C_{15}H_{41}O_6PSi_4$	**2.42**
**10**	13.45	Methyl galactoside	96.4	482	4 TMS	$C_{19}H_{46}O_6Si_4$	**1.87**
**11**	15.45	Palmitic acid	94.3	328	1 TMS	$C_{19}H_{40}O_2Si$	**18.07**
**12**	17.32	Linoleic acid	94.0	352	1 TMS	$C_{21}H_{40}O_2Si$	**2.47**
**13**	17.61	Stearic acid	95.2	356	1 TMS	$C_{21}H_{44}O_2Si$	**4.99**
**14**	18.12	2-O-Glycerol-.alpha.-d-galactopyranoside	95.2	686	6 TMS	$C_{27}H_{66}O_8Si_6$	**16.32**
**15**	19.14	2-Oleoylglycerol	84.9	498	2 TMS	$C_{27}H_{56}O_4Si_2$	**-**
**16**	19.42	3β.24-5-Cholene	80.2	504	2 TMS	$C_{30}H_{56}O_2Si_2$	**4.24**

Identified compounds in the derivatized *A. platensis* ethanolic extract (ES) by GC–MS. with corresponding retention times. molecular formulas. and relative abundance expressed as area percentages

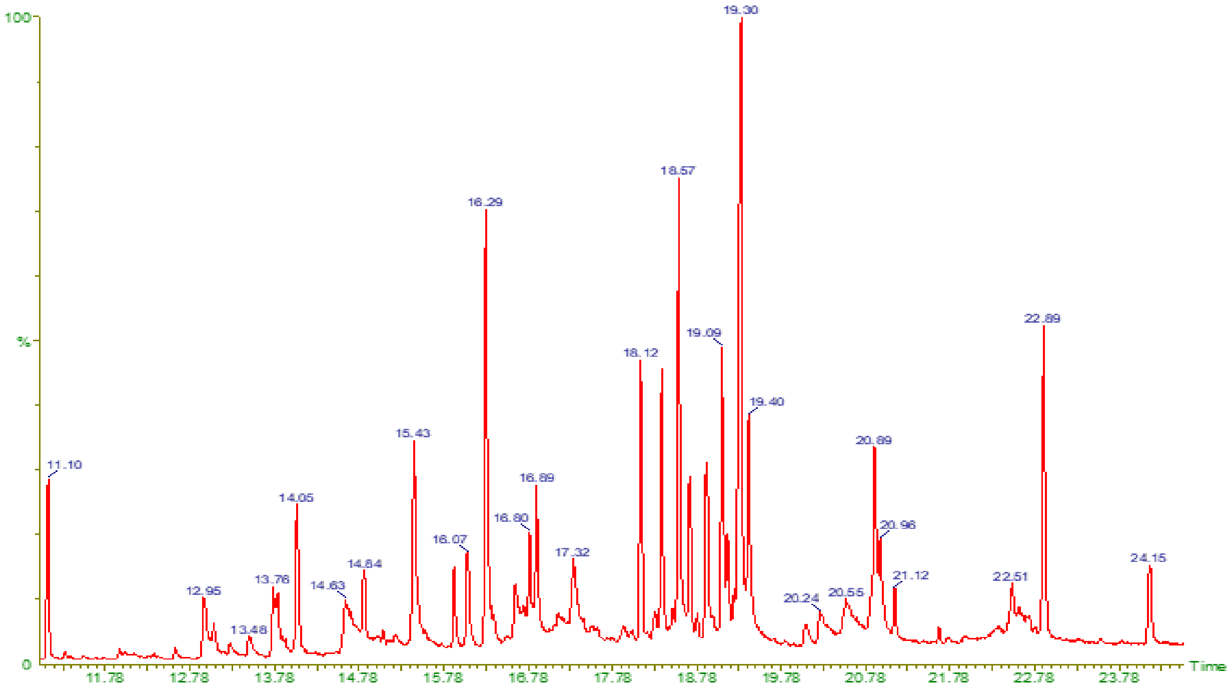

Figure S2. GC-MS chromatogram of the derivatized *A. platensis* aqueous extract (AS).

Table S2. Chemical profile of the derivatized *A. platensis* aqueous extract (AS) identified by GC-MS

Peak	RT (min)	Compounds	Match Score (%)	Molecular Weight (Da)	TMS Derivative	Molecular formula	Area %
**1**	11.10	Hexacosane	99.6	366	-	$C_{26}H_{54}$	**3.68**
**2**	13.76	Dimethyl Palmitamine	97.8	269	-	$C_{18}H_{39}N$	**1.97**
**3**	14.05	Methyl pentadecanoate	99.4	256	-	$C_{16}H_{32}O_2$	**3.63**
**4**	15.43	Palmitic Acid	91.4	328	1 TMS	$C_{19}H_{40}O_2Si$	**7.93**
**5**	16.29	Phytol	97.1	296	-	$C_{20}H_{40}O$	**11.23**
**6**	16.89	Distearyl phosphate	93.8	602	1 TMS	$C_{36}H_{75}O_4P$	**3.28**
**7**	18.12	2-O-Glycerol-.alpha.-d-galactopyranoside	96.0	686	6 TMS	$C_{27}H_{66}O_8Si_6$	**5.89**
**8**	18.57	D-Lyxopyranose	85.0	438	4 TMS	$C_{17}H_{42}O_5Si_4$	**8.99**
**9**	19.09	Levoglucosan	80.0	378	3 TMS	$C_{15}H_{34}O_5Si_3$	**-**
**10**	19.30	D-Xylopyranose	90.1	438	4 TMS	$C_{17}H_{42}O_5Si_4$	**14.67**
**11**	19.40	Methyl galactoside	86.7	482	4 TMS	$C_{19}H_{46}O_6Si_4$	**4.03**
**12**	20.89	Palmitic acid 4-[(trimethylsilyl)oxy]butyl ester	80.0	400	1 TMS	$C_{23}H_{48}O_3Si$	**0.66**
**13**	21.12	2.3-Dihydroxypropyl icosanoate	86.5	530	2 TMS	$C_{29}H_{62}O_4Si_2$	**-**
**14**	22.51	2-Oleoylglycerol	85.9	500	2 TMS	$C_{27}H_{56}O_4Si_2$	**2.29**
**15**	22.89	*α.α'-Trehalose*	84.7	918	8 TMS	$C_{36}H_{86}O_{11}Si_8$	**8.08**
**16**	24.51	Turanose	83.0	918	8 TMS	$C_{36}H_{86}O_{11}Si_8$	**2.60**

Table S3. Original uncropped histological sections

	**G × 40**	**G × 100**
**Control**	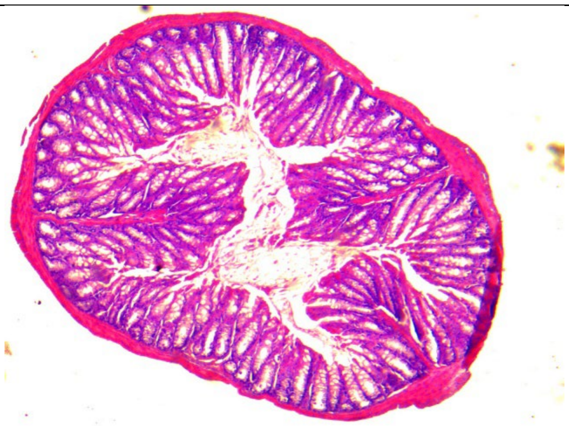	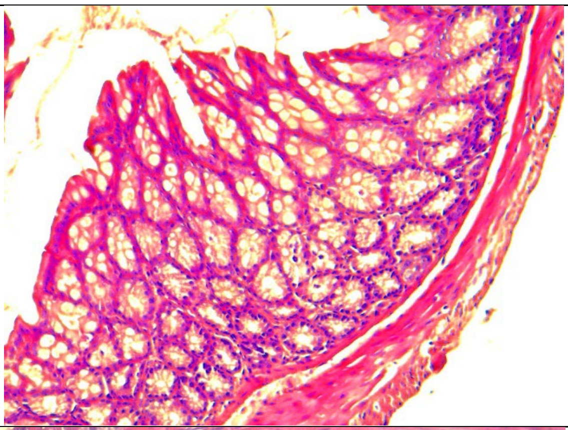
**DNBS**	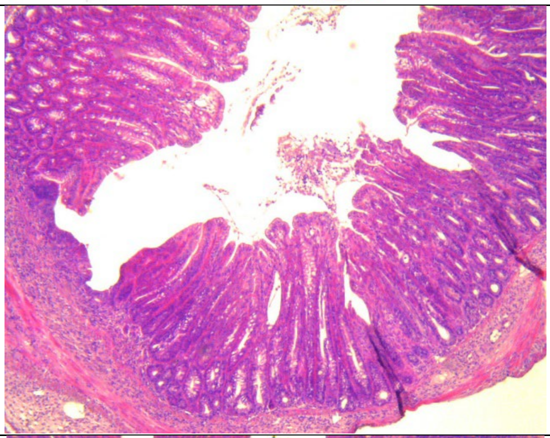	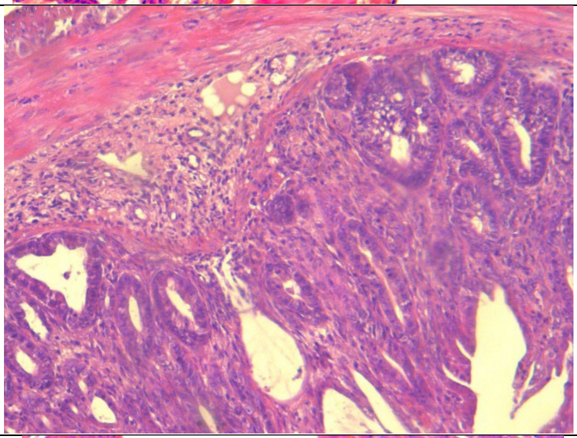
**Dox**	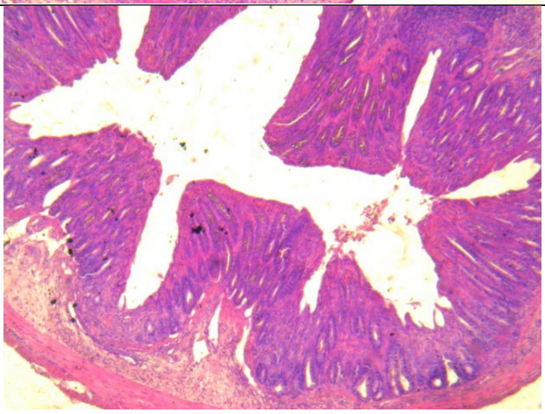	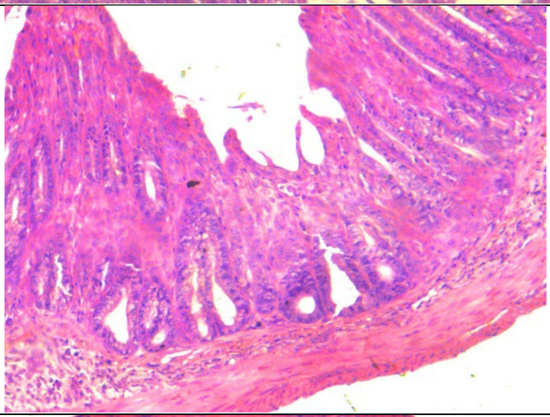
**ES**	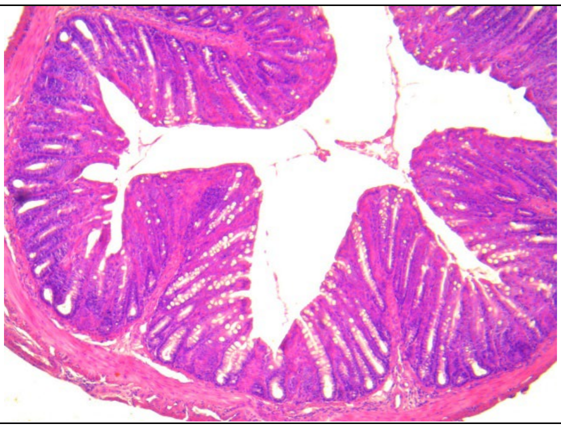	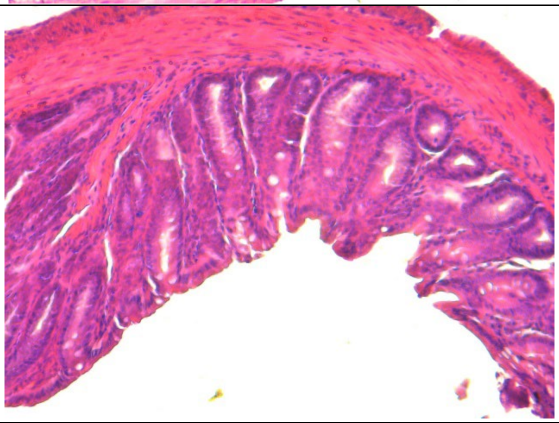

**Dox-ES**	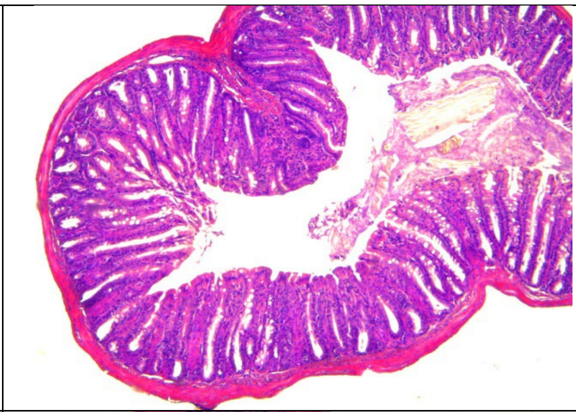	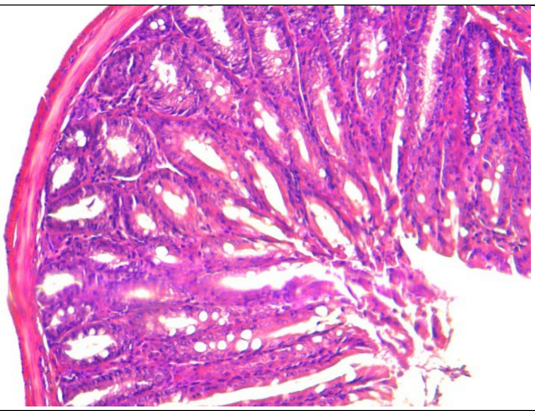
**AS**	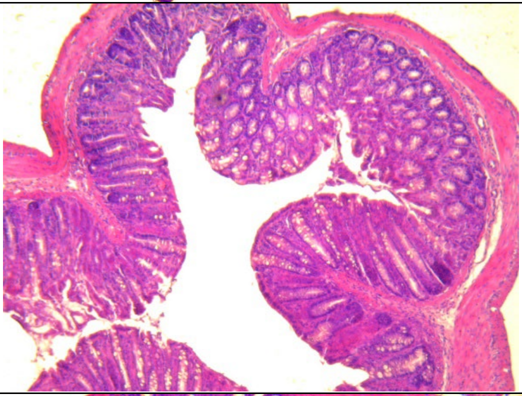	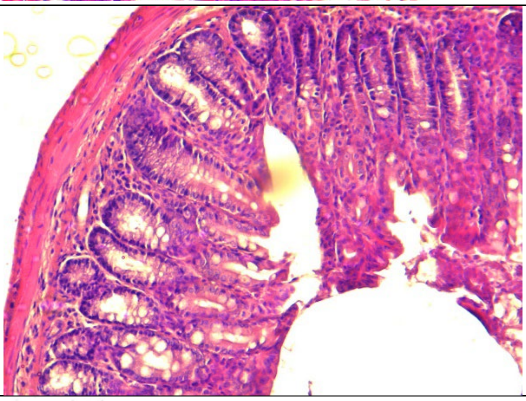
**Dox-AS**	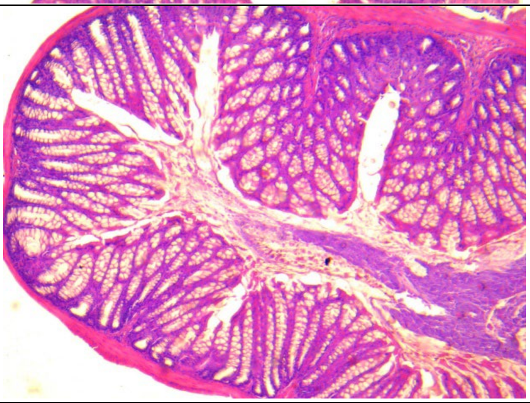	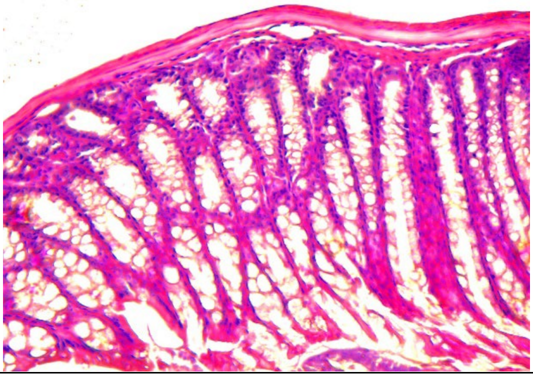
